# Comprehensive Identification of Guan-Xin-Shu-Tong Capsule via a Mass Defect and Fragment Filtering Approach by High Resolution Mass Spectrometry: In Vitro and In Vivo Study

**DOI:** 10.3390/molecules22061007

**Published:** 2017-06-16

**Authors:** Xun Gao, Jingqing Mu, Qing Li, Shaoyi Guan, Ran Liu, Yiyang Du, Huifen Zhang, Kaishun Bi

**Affiliations:** 1 School of Traditional Chinese Medicine, Shenyang Pharmaceutical University, 103 Wenhua Road, Shenyang 110016, China; gaoxun-509@163.com; 2 School of Pharmacy, Shenyang Pharmaceutical University, 103 Wenhua Road, Shenyang 110016, China; mujingqing@126.com (J.M.); lqyxm@hotmail.com (Q.L.); liuran8515@hotmail.com (R.L.); 13897908149@163.com (Y.D.); 3 The General Hospital of Shenyang Military, 83 Wenhua Road, Shenyang 110016, China; guan_shaoyi@126.com

**Keywords:** acute blood stasis, compound, Guan-Xin-Shu-Tong capsule, hyperlipidemia, identification, Q-TOF/MS

## Abstract

The Guan-Xin-Shu-Tong capsule (GXSTC) is a well-known traditional Chinese medicine that is used for the treatment of coronary heart disease. Despite its common use in China, basic pharmacological research on its active components is limited. A comprehensive analytical method using quadrupole-time-of-flight mass spectrometry (Q-TOF/MS), specifically with the Triple TOF 5600 platform, was developed to characterize the compounds in the GXSTC powder itself (in vitro) as well as the active components in healthy and heart disease model rats after its oral administration (in vivo). The 5600 platform was operated in both positive and negative ion modes, before the raw data were processed using the extracted ion chromatography (EIC), mass defect filtering (MDF) and fragment filtering (FF) techniques. With the aid of reference compounds for retention time and fragment ion comparisons, 18 compounds were unambiguously identified in vitro. An additional 56 other compounds were tentatively characterized using the accurate quasi-molecular ion mass and Tandem mass spectrometry (MS/MS) fragmentation pattern strategies. Among them, 30 compounds were characterized based on the MDF and FF approaches. Normal rats in addition to hyperlipidemic (HL) and acute blood stasis (ABS) model rats were given a single oral dose of GXSTC solution for subsequent blood analysis at 1 and 2 h after administration. A total of 24 prototypecomponents and 20 metabolites derived from GXSTC were differentially detected across the three animal groups, including the absence of four phase II phenolic acid metabolites in the ABS group and the presence of three diterpenoid-related metabolites exclusive to the HL group. The use of reference compounds as well as the mass defect and fragment-filtering strategies were critical to identify GXSTC compounds in vitro and in vivo. This can be used for further quality control and pharmacological studies aimed at characterizing the active and potential beneficial compounds of this ancient medicine.

## 1. Introduction

The developments of traditional Chinese medicine (TCM) are based on the clinical outcomes with a history of more than 3000 years [1,2,3,4,5]. With the development of analytical technology and pharmacology, the mechanism of how different ingredients in the formulation work together has attracted more attention than ever before [6,7]. To date, most of the individual materials used in TCM have been investigated for the active compounds [8,9,10]. However, the extracted active compounds may cause additional unexpected side effects compared to the formulation in clinical trials [11,12]. Therefore, it is worthwhile to study the formulation as a whole family, especially with the comparison of the data in vitro and in vivo [13].

The Guan-Xin-Shu-Tong capsule (GXSTC), developed from a Mongolian medicine formulation, has been widely used for the treatment of coronary heart disease and angina pectoris since 2002 in China. It is consisted of *Choerospondias axillaris*, *Salvia miltiorrhiza Bunge*, *Syzigium aromaticum*, *Dryobalanops* and *Tabaschir* at the ratio of 16:8:2:1:1 by weight, with the curative clinical effects on alleviating the degree and scope of myocardial ischemia, minimizing the occurrence of cardiac infarction markedly [14], inhibiting the elevation of phosphate kinase in dogs [15] and decreasing the ventricular premature beat ventricular tachycardia in rats [16]. Furthermore, GXSTC was also found to be effective in the treatment of Alzheimer’s disease [17]. However, the mechanisms of the various ingredients work together at the molecular level and the key active components in vivo are unknown, resulting in the research outcomes not being recognized internationally. Therefore, it is of great importance to investigate its chemical constituents both in vitro and in vivo in order to reveal the real mechanism with the aid of state-of-the-art technologies.

To date, a number of studies on the composition of the single herbs in GXSTC have been reported [18,19,20,21,22,23]. The pharmacological and pharmacodynamics studies [24,25,26,27,28] indicated that the total flavonoids, gallic acid (*Choerospondias axillaris*), diterpenoids and phenolic acids (*Salvia miltiorrhiza Bunge*) in the raw materials may be the main active component. However, only salvianolic acid B, tanshinone IIA, and bornel were used as marker compounds for the quality control in the current specification for GXSTC [29].The chemical substances are one of the important factors to explore their mechanism of action. However, the constituents in GXSTC in vitro and in vivo have not been fully investigated [30]. Until now, there were only three published papers on the chromatographic fingerprint and determination of several compounds in GXSTC by high performance liquid chromatography [31,32] or gas chromatography [33]. Among the five herbs in GXSTC, *Choerospondias axillaris* accounts for the highest ratio (57%). However, there was only nuclear magnetic resonance spectroscopy data available for the purpose of identification of this compound [18,19]. No relevant mass spectrometry data have been reported and a similar situation was found for *Tabaschir*. The reported identification analysis of *Salvia miltiorrhiza Bunge* only included nuclear magnetic resonance spectroscopy, high performance liquid chromatography and liquid chromatography quadrupole-time-of-flight mass spectrometry (LC-Q-TOF/MS) [20,21,22,23]. Currently, LC-Q-TOF/MS has been applied widely in the separation and identification of complex components in TCM [30,34,35]. Compared to other mass spectrometry methods, Q-TOF/MS has the characteristics of greater accuracy, higher scanning speed and a wider range of masses [36]. It can provide an accurate molecular mass and adequate structural information for the characteristic fragment as well as the isotopic abundance and element composition [37]. For mass data provided by high-resolution mass spectrometry, the information dependent acquisition (IDA) strategy can maximize the discovery of compounds [30].Mass defect refers to the difference between the exact molecular weight of an element (compound) and its nearest integral value. For the similar structure compounds (such as the parent drug and its metabolites being natural homologues), there is a large difference in the molecular weight of integral part, but the mass defect is usually located in a very narrow range [35]. This provides the theoretical basis for identification of a type of compounds with similar structure via mass defect filtering (MDF). For TCM, a type of compounds with similar structure usually occurs in a similar cleavage in collision-induced dissociation to generate one or more similar fragment ions. At the same time, the neutral loss of specificity is common in the collision induced dissociation process, such as the loss of neutral sugar residue in the most common glycosides [38]. MDF and FF approaches can greatly reduce the chromatogram response of non-target compounds and back ground ions, so that the covered target compounds in complex background could appear and be qualitatively analyzed [39,40].

In this paper, a comprehensive analytical method with high resolution, sensitivity, and accuracy based on Q-TOF/MS was developed to explore the compounds in the GXSTC powder itself (in vitro) and the active components of GXSTC in vitro for the first time. Furthermore, the data profiling procedure with the MDF and FF approaches was highlighted in this study. There were mainly three steps for this study design strategy. The first step was to characterize the compounds in GXSTC (in vitro) and further identify their source by comparing them with five plant extracts, respectively. Then, the typical heart disease models of rats were established in order to approach the clinical condition, which included hyperlipidemia (HL) and acute blood stasis (ABS) models. The last step was to identify and statistically analyze the active components in healthy and heart disease model rats after oral administration of GXSTC (in vivo).This study may be the foundation for the quality control and pharmacological study for GXSTC, which may further provide valuable data for the development of new drug.

## 2. Results and Discussion

### 2.1. Optimization of Method Conditions

The acetonitrile/water system provided better separation, sensitivity and less background noise when compared with methanol/water system. Furthermore, the addition of formic acid with different ratios (from 0.01% to 0.5%) could inhibit ionization of acidic ingredients in the sample and influence the peak shape. Overall, 0.1% formic acid acetonitrile/water was the optimal solvent systems in this paper.

### 2.2. Analysis of the Compounds of GXSTC In Vitro

**Figure 1:**
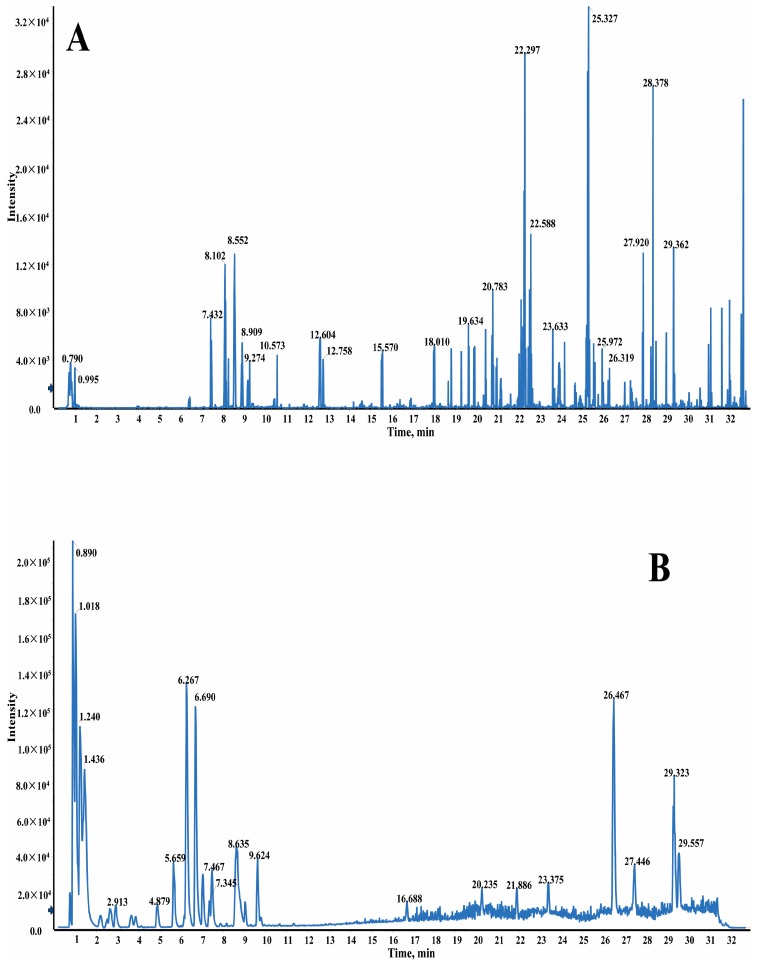

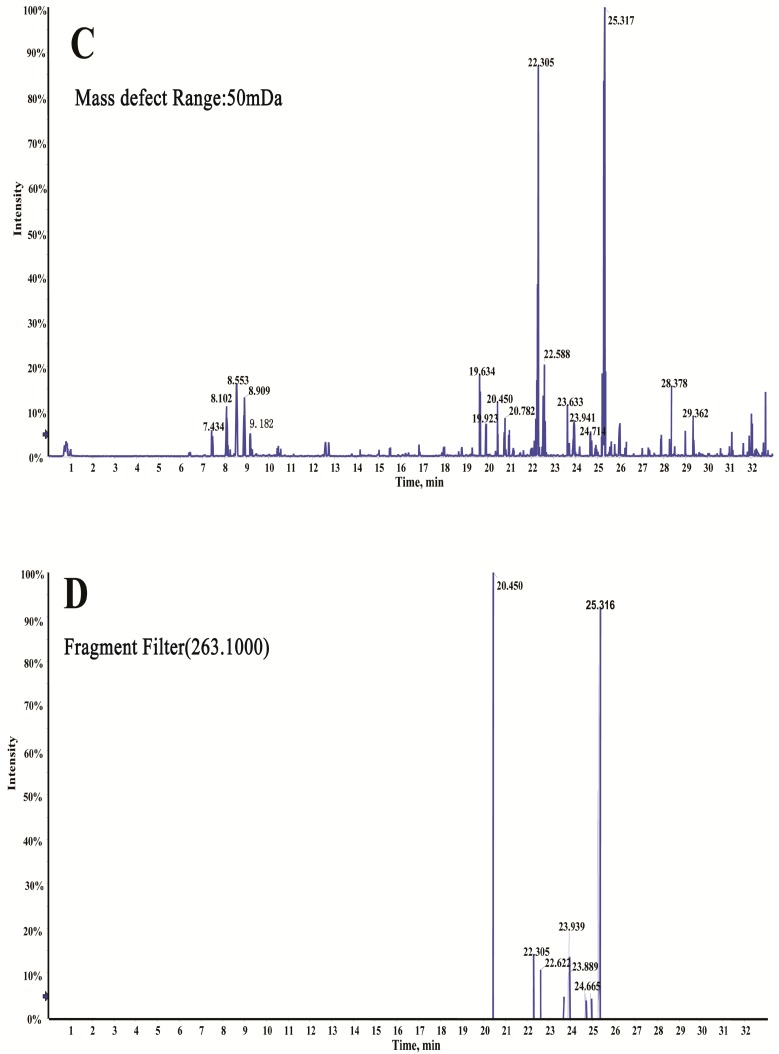
Base peak chromatograms (BPCs) of GXSTC in positive mode (**A**) and in negative mode (**B**) by liquid chromatography quadrupole-time-of-flight mass spectrometry (LC-Q-TOF/MS), and total ion chromatograms after MDF (**C**) and FF (**D**) in positive mode.

Under the optimized LC–MS conditions described above, the base peak chromatograms (BPC) of GXSTC in positive and negative modes in vitro are shown in Figure 1A,B. A total of 18 compounds were unambiguously identified by comparing the retention time and MS data against that of the reference standards, which are illustrated in Table S1. The other 56 compounds were tentatively characterized by an accurate mass of quasi-molecular ions (<5 ppm) and MS/MS fragmentation patterns with the aid of the related literature. Among them, 30 compounds were identified based on MDF and FF approach, which are shown in Table 1. A total of 74 compounds were identified in GXSTC in vivo, which is listed in Table 2 with the ion fragment MS/MS data in Table S2. Furthermore, the structures of 74 compounds are shown in Figure S1.

#### 2.2.1. MDF Approach Profiling

The efficient and reliable MDF has a powerful data processing capability. For example, the theoretical mass defect of 0.1557 Da of tanshinone IIA was adopted as the mass defect filtering reference or preliminary screening of diterpenoids. The defect tolerance and the filtering mass range were set at 50 mDa and 200–400 Da, respectively. Following this, a new chromatogram via MDF was generated and shown in Figure 1C, which possessed fewer peaks for target and systematical analysis.

#### 2.2.2. Fragment Filtering Approach Profiling

After unambiguous identification of cryptotanshinone, tanshinone I, dihydrotanshinone I, tanshinone IIA and miltirone by comparing the retention time and MS/MS data with the corresponding reference standard, the fragment ions of theoretical values for mass-to-charge ratio (*m*/*z*) 279.1, 265.1, 263.1, 261.1 and 233.1 were found to be the typical fragment ions for diterpenoids, which were selected as the referencesin the FF. The predicted compound of dehydrotanshinone was used to demonstrate the method of identifying the compound with the filter. When the fragment of *m*/*z* 263.1000 was taken into the fragment filter and employed in Experiment 2, a filtered chromatogram was generated (Figure 1D). The peaks with retention times of 22.31 min and 25.32 min were already identified preliminarily as cryptotanshinone and tanshinone IIA by target screening, while the peaks with retention times of 20.45 min, 23.94 min and 24.67 min were listed as unknown compounds to be identified. The collision-induced dissociation information from the quadrupole shown only a rough precursor ion *m*/*z* of 293.1 and an accurate mean *m*/*z* of 293.1181 was obtained from the TOF/MS in accordance with the retention time. The formula was predicted as C_19_H_16_O_3_ with the aid of the formula finder function, provided by PeakView^®^ software (version 2.2, Sciex, Redwood City, CA, USA). After screening the identified diterpenoids, tanshinone IIA was found to have fragments of *m*/*z* 178.0771, 219.1169 and 235.0750 similar to the unknown compound. Consequently, the compound was predicted as 1-dehydrotanshinone with the aid of the literature [41]. The other two compounds were predicted as 1-dehydromiltirone and danshexinkun D by a similar FF approach.

#### 2.2.3. Identification of Flavonoids and Their Glycosides

**Figure 2:**
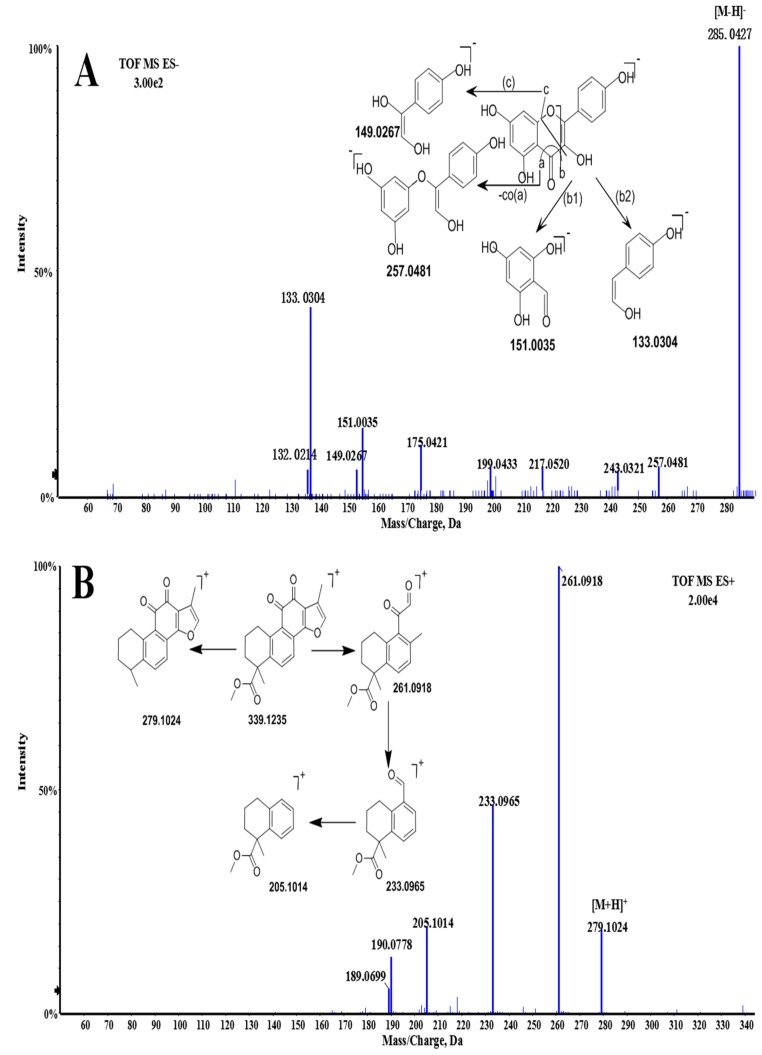

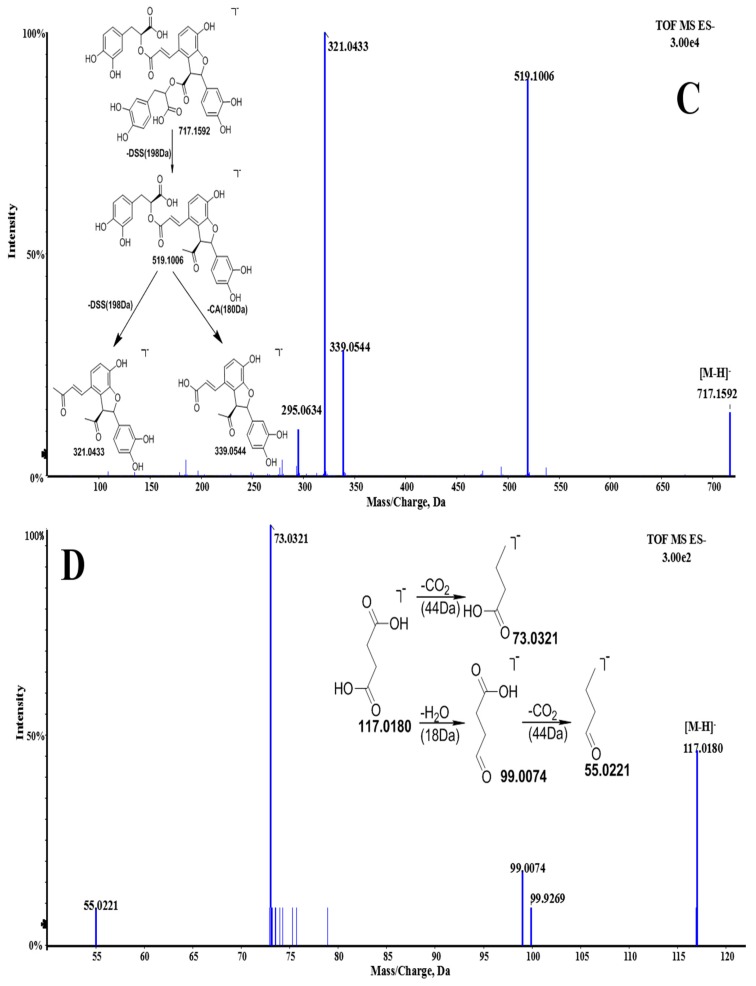
The MS/MS spectra and proposed fragmentation pathways of kaempferol (**A**); methyltanshinonate (**B**); salvianolic acid B (**C**); and succinic acid (**D**).

The total flavonoids in the raw *Choerospondias axillaris* were reported to be the most significant compounds that might be associated with the pharmacological effects in the treatment of cardiovascular diseases [42,43,44]. A total of 7 types of flavonoids and their glycosides were identified in vitro. Among them, compound **31** was unambiguously identified as quercetin by comparison of the retention time and TOF/MS data with its reference standards. The MDF and FF approaches were applied in the flavonoid identification. The mass defect of 0.1137 Da (kaempferol) was selected as the MDF reference along with the defect tolerance of 50 mDa and the filtering mass range of 200–600 Da. Through the analysis of flavonoid fragmentation, the fragments of *m*/*z* 133.0 and *m*/*z* 151.0 in the negative ion mode in addition to *m*/*z* 301.0 in the positive ion mode were adopted as FF references. Compound **30** (kaempferol) was selected as the example to demonstrate the fragmentation pathways (Figure 2A). It showed a molecular ion [M − H]^−^ at *m*/*z* 285.0427, which may further lose CO (28Da) to form the fragment with an *m*/*z* 257.0481. The ions at *m*/*z* of 151.0035 and 133.0304 were due to the loss of C_8_H_6_O_2_ and C_7_H_4_O_4_ group from the parent ion. In addition, compound **16** (hyperin) showed a molecular ion [M + H]^+^ at an *m*/*z* of 465.1133 in the positive mode and product ion at an *m*/*z* of 301.0438, indicating the loss of galactopyranose from the precursor ion sequentially, which was the same as compound **19** (kaempferol-7-*O*-glucopyranoside). 

#### 2.2.4. Identification of Diterpenoids

Diterpenoids may be the most important lipid-soluble bioactive constituents and reference standards in the quality control of *Salvia miltiorrhiza Bunge* [45,46]. They generally produce abundant molecular ions of [M + H]^+^ in the positive mode and generate the fragmentations with the loss of the methyl group, hydroxy group, carbon monoxide or carbon dioxide radical. In this study, 25 types of diterpenoids [10,41,47] were detected in GXSTC. Taking compound **55** (methyl tanshinonate) as an example (Figure 2B), a molecular ion [M + H]^+^ at an *m*/*z* of 339.1236 has been observed in the positive mode. The ion spectrum at an *m*/*z* of 279.1024 (MS/MS) suggested the loss of one methyl group and one carboxyl, which may be due to the loss of H_2_O, CO and 2CO, resulting in the fragments at *m*/*z* of 261.0918, 233.0965 and 205.1014, respectively. 

#### 2.2.5. Identification of Phenolic Acids

The phenolic acid may be the most important hydrophilic bioactive constituents and reference standards in the quality control of *Salvia miltiorrhiza Bunge* [46,48].

In vitro, a total of 23 phenolic acids [49,50,51] were detected in GXSTC and twelve of them were further confirmed by MS/MS data against their reference standards, including gallic acid, danshensu, protocatechuic acid, procatechuic aldehyde, caffeic acid, ellagic acid, salvianolic acid B, salvianolic acid A, salvianolic acid C, chlorogenic acid, rosmarinic acid and eugenol. They usually contain two or more phenyls in their structures, which result in the fragmentations of danshensu (DSS), caffeic acid (CA) and caffeoyl due to the loss of C_9_H_10_O_5_, C_9_H_8_O_4_, and C_9_H_6_O_3_, respectively [49]. As shown in Figure 2C, salvianolic acid B was selected to investigate the fragmentation pathways. It exhibited fragment ions at *m*/*z* of 717.1592 [M − H]^−^, 519.1006[M-C_9_H_10_O_5_(DSS)-H]^−^, 339.0544 [M-C_9_H_10_O_5_(DSS)-C_9_H_8_O_4_(CA)-H]^−^ and 321.0433[M-C_18_H_20_O_10_(2DSS)-H]^−^. The MDF and FF approacheswere applied in the phenolic acid identification. The mass defect of 0.2328 Da (lithospermic acid) was selected as the MDF reference along with the defect tolerance of 50 mDa and the filtering mass range of 400–600 Da. The fragments of *m*/*z* 313.0 and *m*/*z* 367.0 in the negative ion mode were adopted as FF references.

#### 2.2.6. Identification of Organic Acid

In this study, 6 organic acids derived from *Choerospondias axillaris* were detected in vitro. Compound **9** (Figure 2D) showed a molecular ion [M − H]^−^ at an *m*/*z* of 117.0180 in the negative mode. The ions at *m*/*z* of 99.0074 and 73.0321 by MS/MS spectrum suggested the loss of one H_2_O group (18Da) and carbon dioxide (44 Da). By comparing exact molecular mass and MS/MS spectra with the published data [20], it was tentatively identified as succinic acid. Following this, the MDF parameters were set as follows: 0.0672 Da (succinic acid) for mass defect, 50 mD for the defect tolerance and 100–300 Da for the filtering mass range. The fragments of *m*/*z*151.0, *m*/*z* 99.0and *m*/*z* 73.0 in the negative ion mode were adopted as the FF references.

#### 2.2.7. Others

Other compounds originating from GXSTC in vitro were tentatively identified as amino acids, alkaloids, steroidal saponins and coumarins based on the MDF and FF approaches (Table 1).

With the developed rapid method and according to the following screening principles thatwere suggested: (1) the compounds should be closely related to the clinical efficacy and may be sensitive to the production procedure; (2) the unique compound belonging to each single raw material has no interference from the other materials in the identification; (3) the compound account for a comparatively high or low ratio level in the formulation; (4) the reference standard of the ingredients should be easy to access. A total of 40 compounds (7 flavonoids and their glycosides, 15 diterpenoids, 12 phenolic acids, 2 organic acids and 1 steroidal saponin as well as eugenol and its derivatives) were proved to exist in GXSTC and were considered to be the potential candidates of the marker compounds for the quality control in future specification or chromatographic fingerprint common peak attribution of GXSTC.

**Figure 3:**
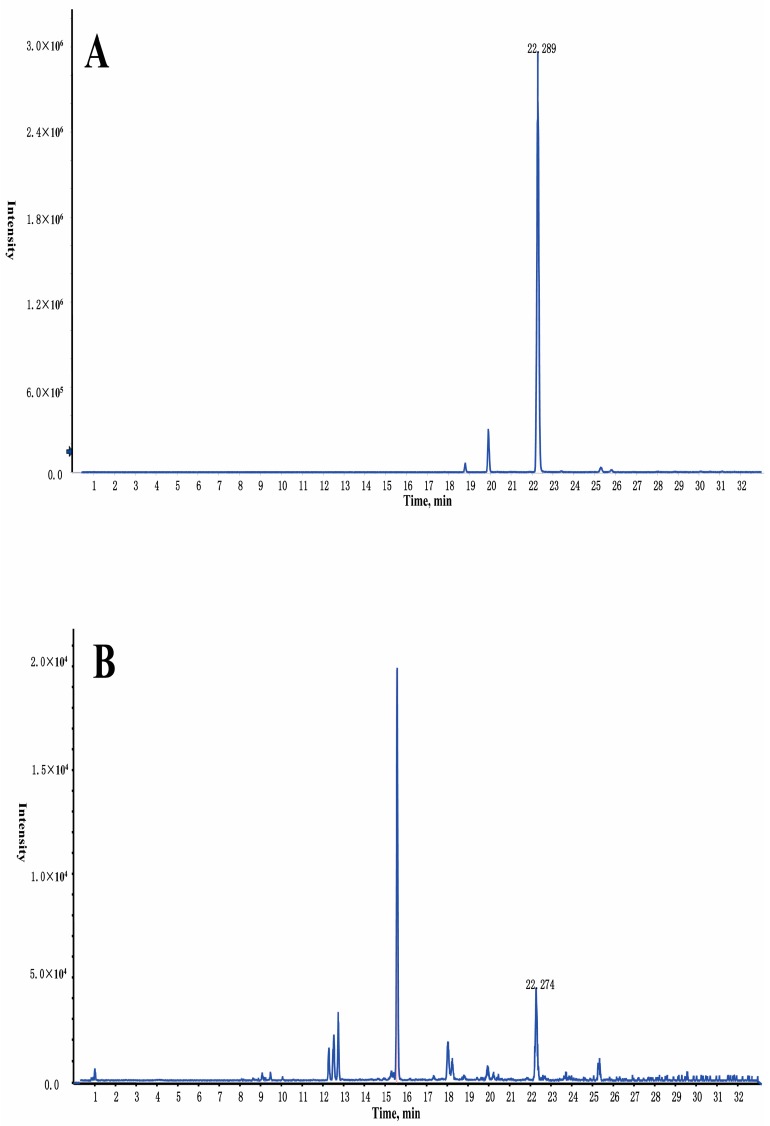
The LC-Q-TOF/MS extracted ion chromatographs (EIC) of cryptotanshinone in GXSTC (**A**) and *Salvia miltiorrhiza Bunge* (**B**) in the positive ion mode.

In addition, the five materials (*Choerospondias axillaris*, *Salvia miltiorrhiza Bunge*, *Syzigium aromaticum*, *Dryobalanops and Tabaschir*) in GXSTC were analyzed individually to identify the source of the compounds in Table 2. Interestingly, some compounds were found in the capsule compared with a single herb. Taking cryptotanshinone as an example, its intensity significantly increased (*p* < 0.05) in GXSTC samples (Intensity = 2968052）compared to the data obtained from the individual *Salvia miltiorrhiza Bunge* (Intensity = 19815), which is shown in Figure 3.

### 2.3. Analysis of the Active Component of GXSTC In Vivo

#### 2.3.1. Analysis of the Prototype Components of GXSTC In Vivo

HL is an important cause of coronary heart disease and the HL rat model is the classical model of early coronary heart disease [52]. The ABS is closely related to coronary heart disease [53,54]. In this paper, there were normal, HL, and ABS groups used to investigate the difference in serum pharmaceutical chemistry at different stages of coronary heart disease. According to the literature [12,13,55], the *T*_max_ of diterpenoids and phenolic acids in *Salvia miltiorrhiza Bunge* ranged from 1 h to 2 h, so the blood samples at 1 h and 2 h time points were collected in this paper. However, there was no significant difference between the 1 h and 2 h for active components in healthy and heart disease model rats after oral administration of GXSTC.

The BPC of normal groups, ABS groups and HL groups in both modes are presented in Figures S2–S4. In vivo, 44 peaks were detected in dosed plasma but not in control plasma. Among these peaks, 20 peaks also appeared in the MS spectra of GXSTC, indicating that these components were absorbed into the rat plasma in the original form. Based on the retention times and the accurate MS/MS fragment data, 24 compounds as prototype compounds were identified both in vitro and in vivo (Table 3) with their chemical structures in Figure S1,including dihydroquercetin and 9 diterpenoids (tanshindiol A/C, tanshinone IIB/hydroxytanshinone IIA, cryptotanshinone, tanshinone I, dihydrotanshinone I, tanshinone IIA and miltirone), 8 phenolic acids (gallic acid, danshensu, protocatechuic acid, caffeic acid, rosmarinic acid, salvianolic acid B, chlorogenic acid and 3,3′-di-*O*-methylellagic acid), 5 organic acids (malic acid, citric acid, stearic acid, linoleic acid and palmic acid) and betaine.

#### 2.3.2. Analysis of the Metabolites of GXSTC In Vivo

The analysis of the prototype components and their metabolites in the plasma could be the fundamental data for the identification of the active compounds in TCM [12,13].

**Figure 4:**
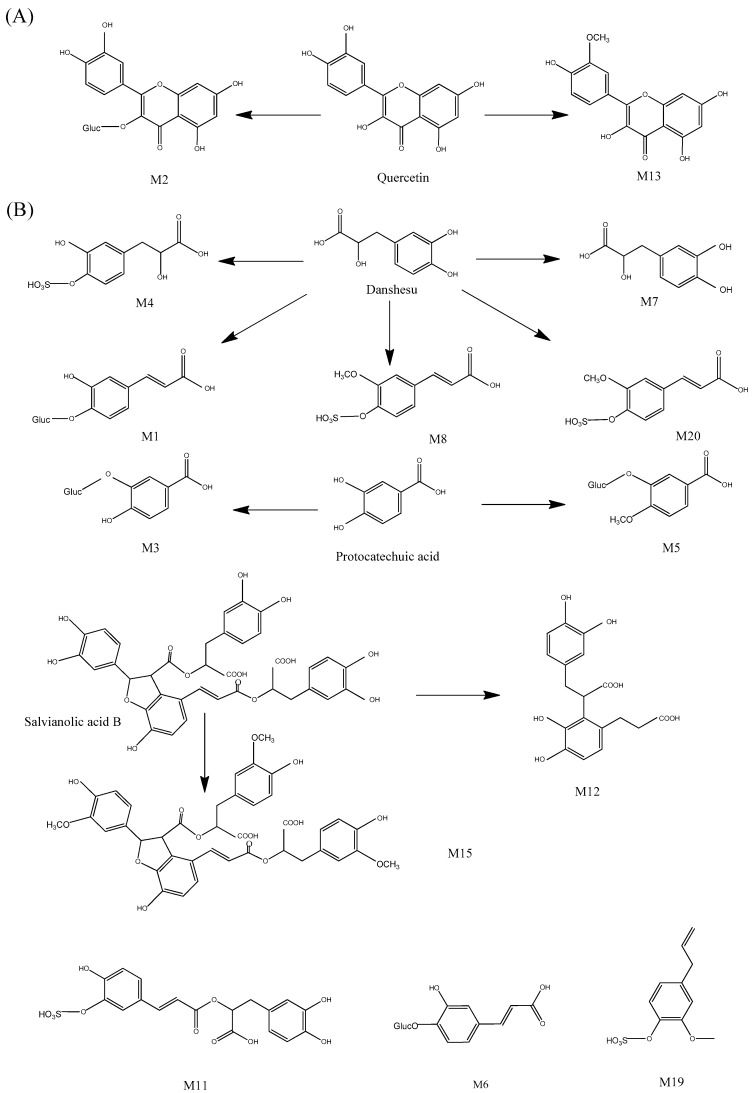

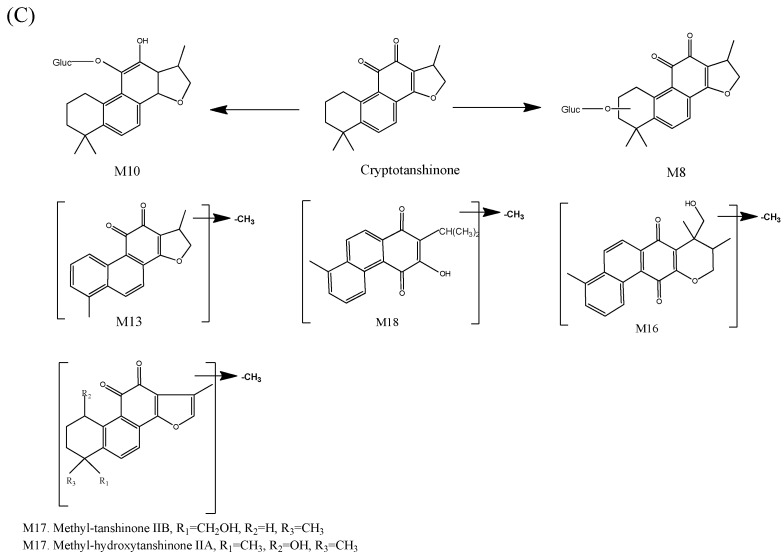
The proposed metabolic pathways of flavonoid-related metabolites (**A**); phenolic acid-related metabolites (**B**); and diterpenoid-related metabolites (**C**) in rat plasma.

**Figure 5:**
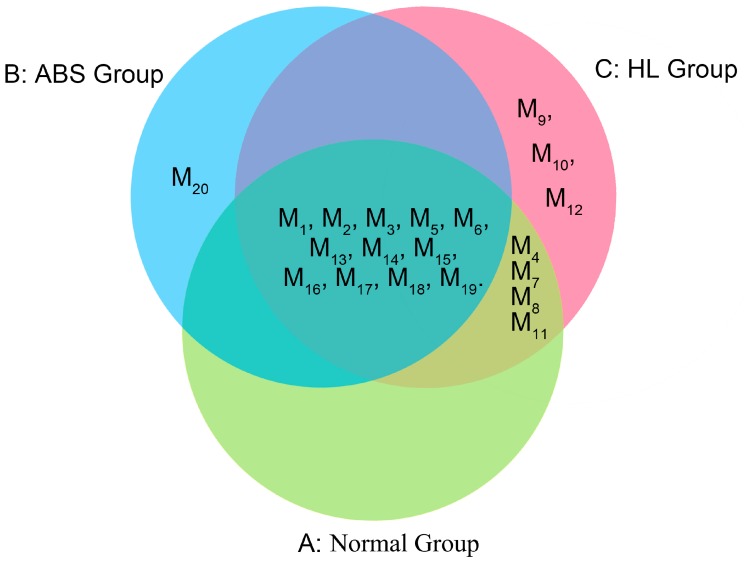
The Venn diagram of all metabolites in the Normal group (**A**); the ABS group (**B**); and the HL group (**C**).

Compounds detected only in the rat plasma after oral administration of GXSTC may be the degraded substances or endogenous metabolites. Based on the MDF and FF approach, 20 metabolites were tentatively identified in rat plasma after oral administration (Table 4) with the proposed metabolic pathways in Figure 4 .In order to make a thorough comparison of their derivatives or metabolites in the normal group, HL group and ABS group, each of the above-mentioned chemical categories was fully discussed. Furthermore, a Venn diagram of all metabolites was clarified for all groups as shown in Figure 5. Among them, M1, M2, M3, M5, M6, M13, M14, M15, M16, M17, M18 and M19 were detected in the normal group, while the pathological model groups. M4, M7, M8 and M11 were detected in normal and HL groups. However, M9, M10 and M12 were only detected in HL group, while M20 was only detected in ABS group.

##### Identification of Flavonoid-Related Metabolites

M2 and M13 were detected in rat plasma as the flavonoid-related metabolites after oral administration of GXSTC. M2 and M13 exhibited an accurate molecular ion of [M − H]^−^ at an *m*/*z* of 477.0665 and 315.0524, respectively. After screening the identified flavonoids, quercetin was found to have similar fragments of *m*/*z* of 275.0561, 177.0193 and163.0037 to the unknown compounds. Consequently, this produced fragments of 176 Da and 14 Da higher than that of quercetin, respectively. M2 and M13 were tentatively identified as quercetin-3-*O*-glucoside and quercetin-3′-methyl ether, respectively. The results showed that the flavonoids experienced methylation and glucuronidation (Phase II) by various drug metabolizing enzymes in the rat after oral administration of GXSTC. Furthermore, the content of the methylated product in the ABS group (M13) increased obviously compared with the other two groups. However, the rats and humans processed food in different ways, so the results proven in rats would be different from those experienced by patients in clinical practice.

##### Identification of Phenolic Acid-Related Metabolites

M1, M3–M8, M11, M12, M15, M19 and M20 are phenolic acid-related metabolites from processes, including dehydrogenate, methylation, glucuronidation and sulfating. M3 showed a molecular ion [M − H]^−^ at an *m*/*z* of 329.0514, which was calculated as being C_13_H_14_O_10_ and being 176 Da (C_6_H_8_O_6_) more than that of protocatechuic acid. The ion at an *m*/*z* of 153.0207 in the negative ion mode indicated a diagnostic neutral loss fragment of glucuronide. In addition, the fragment ion at the *m*/*z* of 109.0369 in the negative ion mode was similar to protocatechuic acid. Therefore, M3 was tentatively identified as protocatechuic acid-glucuronide and similarly, M6 was tentatively identified as CA-glucoside. M5, with an accurate molecular ion [M − H]^−^ at an *m*/*z* of 343.0670, was 14 Damore than that of M3, revealing astructure of methyl protocatechuic acid-glucuronide. M15, with a molecular ion [M − H]^−^ at an *m*/*z* of 759.1925, was 42 Da more than that of salvianolic acid B. M12 (molecular ion [M − H]^−^ at an *m*/*z* of 361.0925),was inferred to be the product of salvianolic acid B with two ester bonds and the furan ring cleavage. Based on the MDF approach and MS/MS spectra fragmentation, they were tentatively identified as trimethyl-salvianolic acid B and salvianolic acid R.

M11 showed a molecular ion of [M − H]^−^ at an *m*/*z* of 439.0340 (80 Da more than rosmarinic acid). By comparing MS and MS/MS spectra, they were tentatively identified as rosmarinic acid-sulfate. In the same methodology, M4, M19 and M20 were tentatively identified as DSS-sulfate, eugenol-sulfate and methyl DSS-sulfate, respectively. M1 showed an accurate molecular ion of [M − H]^−^ at an *m*/*z* of 355.0666, which is 176 Da more than an *m*/*z* of 179.0347. This was produced by the neutral loss of oxygenate and hydrogenation from DSS. M7 showed an accurate molecular ion of [M − H]^−^ at an *m*/*z* of 165.0565 and its formula was calculated as C_9_H_10_O_3_, which was produced by the neutral loss of oxygenate from DSS. With the data of the MS/MS spectra fragmentation and literature [55], M1, M7 and M8 were tentatively identified as dehydrogenate and dehydroxylate DSS-glucuronide, deoxygenate-DSS and dehydroxylate methyl DSS-sulfate.

Interestingly, M4, M7, M8 and M11 were not detected in the ABS group and our pharmacokinetic study (data not shown) showed that the elimination rate for phenolic acid was significantly reduced in this group. This may be due to the ABS-induced decrease in drug elimination [56,57]. Therefore, the absence of the above phase II metabolites of phenolic acid at 1 h and 2 h after administration may be due to the reduced drug elimination rate, weakened re-absorption or alteration of sulfotransferase, which is responsible for the hydroxylation and sulfation [58]. In addition, the content of M5 (methyl protocatechuic acid-glucuronide) in the ABS group was two times higher than the normal and HL groups. Combined with the different contents of M13 in each group mentioned above, it is inferred that the activity of methyltransferase is up regulated in the ABS pathological state for the first time.

Both M11 and M19 are sulfated metabolites of phenolic acids, which are closely related to the activities of sulfotransferase. The content of M11 in the HL group was nearly 4 times higher than that of the healthy group, which was inferred to be caused by the up-regulation of sulfotransferase activity. However, the content of M19 in the HL group was far less than that of the healthy group. Therefore, the specific mechanism is worthy of further study.

##### Identification of Diterpenoid-Related Metabolites

M16 presented an accurate molecular ion of [M + H]^+^ at an *m*/*z* of 351.1601 with 14 Da more than that of danshenxinkun D. The MS/MS fragmentation in the positive ion mode revealed a fragment ion at an *m*/*z* of 291.1016 and 199.0965 as the characteristic ion of danshenxinkun D. Consequently, M16 was tentatively identified as methyl-danshenxinkun D. In the same methodology, M14, M17 and M18 were tentatively identified as methyl-dihydrotanshinone, methyl-tanshinone IIB/methyl-hydroxytanshinone IIA and methyl-danshexinkun B. The above results indicated that the diterpenoid from GXSTC may be methylated by the hepatic microsomal enzyme system and re-absorbed into the bloodstream. 

M10 showed a molecular ion of [M − H]^−^ at an *m*/*z* of 473.1837 and the production at an *m*/*z* of 297.1555 in the MS/MS spectra, which was a diagnostic neutral loss fragment from glucuronide-conjugated compounds. According to a previous study [59], M9 and M10 were tentatively identified as hydroxylated cryptotanshinone-glucoronide and cryptotanshinone catechol-glucuronide. In addition, M9, M10 and M13 were only observed in the HL group. This may be caused by the disorder of the lipid metabolism, absorption pathways [60,61], hydroxylation (Phase I) and glucuronidation (Phase II), which leads to the re-absorption of the metabolites.

##### Statistical Analysis

**Figure 6:**
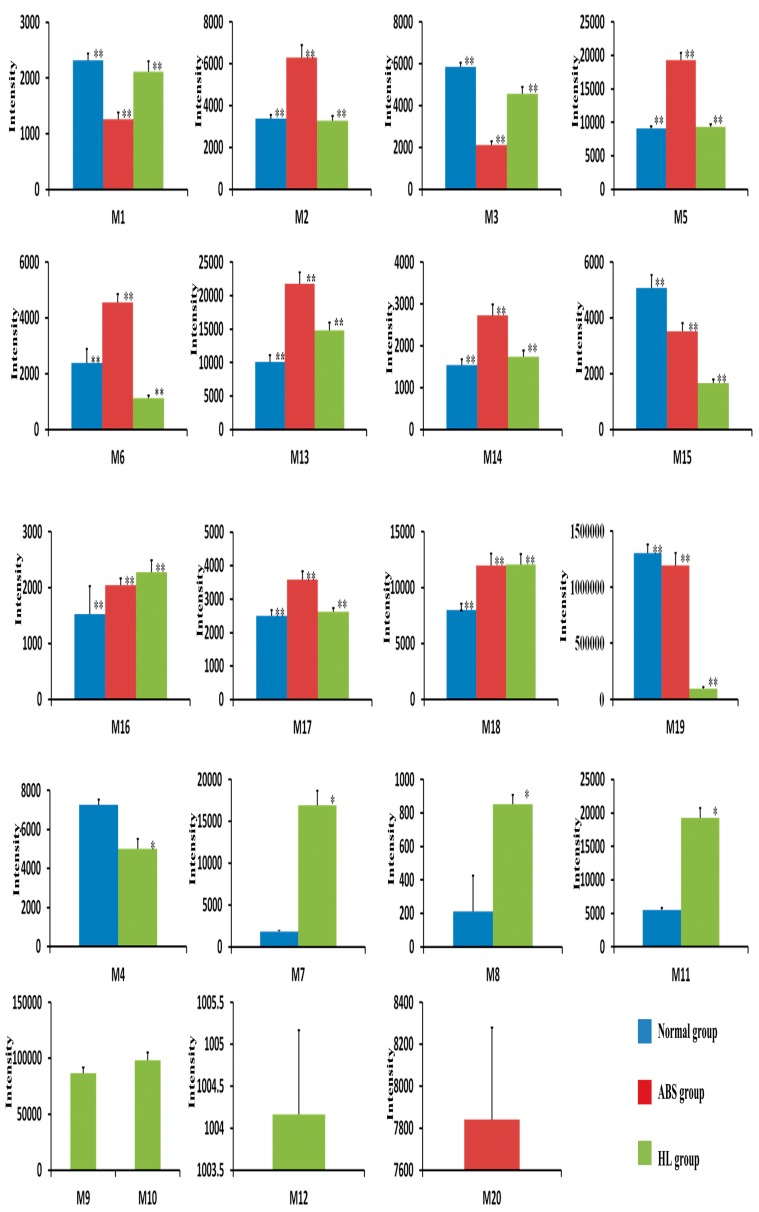
Mean level of 20 metabolites in normal, ABS, and HL groups. The *t*-test was used to investigate the significant difference for each metabolite between two groups (* *p* < 0.05). One-way ANOVA was applied to analyze the significant difference among three groups (** *p* < 0.05).

The results of the statistical analysis of all metabolites from different groups are illustrated in Figure 6. For M4, M7, M8 and M11 in the normal group and HL group, a *t*-test of independent samples was employed to show significant differences (*p* < 0.05). One-way ANOVA was applied to analyze the differences between the normal group, ABS and HL groups. There were significant differences in the metabolites of M1, M2, M3, M5, M13, M14, M15, M16, M17, M18, and M19 in the above groups (*p* < 0.05). The results indicated that the physiological state played a critical role in this process. In addition, for M6, there was no significant difference between ABS and HL groups. However, the significance was observed when comparing the ABS and HL groups to normal group.

## 3. Materials and Methods

### 3.1. Chemicals and Reagents

The GXSTC, *Choerospondias axillaris*, *Salvia miltiorrhiza Bunge*, *Syzigium aromaticum*, *Dryobalanops* and *Tabaschir* were kindly provided by Buchang Pharmaceutical Co. Ltd. (Xi’an, China). The reference standards of gallic acid, danshensu, protocatechuic acid, protocatechuic aldehyde, ellagic acid, rosmarinic acid, salvianolic acid A/B/C, chlorogenic acid, eugenol, dihydrotanshinone I, cryptotanshinone, tanshinone I/IIA, caffeic acid, quercetin and miltionone were purchased from Nantong Feiyu Biotechnology Co. Ltd (Nantong, China). Their mass analysis was performed on the Triple TOF^TM^ 5600 spectrometer (AB SCIEX, Foster City, CA, USA) according to previous literature [18,19,20,21,22,23]. Meanwhile, the purity of all standards was determined to be above 98% by LC analysis based on a peak area normalization method. 

Acetonitrile and methanol (HPLC-grade) were purchased from Fisher Scientific (Fair Lawn, NJ, USA). Formic acid (HPLC grade) and phosphoric acid (analytical grade) were supplied by Kemiou Chemical Reagent Co. Ltd. (Tianjin, China). Deionized water was purified by the Milli-Q water purification system from Millipore (Bedford, MA, USA). All other chemicals and reagents were analytical-grade and commercially available.

### 3.2. Animal Experiment

A total of thirty-six male Sprague-Dawley rats (220–250 g) obtained from the Experimental Animal Center of Shenyang Pharmaceutical University (Shenyang, China) were kept in communal plastic cages for an adaptation period of 7 days with the temperature ranging from 22 °C to 24 °C and a relative humidity of 50–60% before the oral administration. All rats were fasted for 12 h with free access to water prior to the experiment, followed by random separation into six different groups. These groups were normal control, normal dosed, HL control, HL dosed, ABS control and ABS dosed, with six rats in each group. Rats in the HL groups were fed with a high-fat diet (15% lard, 5% custard powder, 2% cholesterol, 1% sodium cholate, 0.2% propylthiouracil and 76.8% standard chow) for 6 weeks. Rats in the ABS groups were induced by left anterior descending branch ligation. The GXSTC powder was dissolved in a 0.5% carboxymethyl cellulose sodium salt (CMC-Na) buffer solution followed by single oral administration at a dose of 5.0 g/kg. For the control group, placebo samples (0.5% CMC-Na solution) were used. Blood samples were collected from the ophthalmic vein at 1 h and 2 h after administration and then centrifuged immediately at 4000 rpm for 10 min at 4 °C to obtain the plasma (TGL-16, Changsha Xiangyi Centrifuge Instrument Co., Ltd., Changsha, China), which was frozen at −80° C until analysis. The animal experimental protocol was approved by the University Ethics Committee for the use of rats. The guidelines for the care and use of laboratory animals were followed for all the animal-related experiments.

### 3.3. Sample Preparation

#### 3.3.1. Sample Preparation for In Vitro Analysis

The GXSTC powder (0.5 g) was extracted with 25 mL of ethanol/water (70:30 *v*/*v*) under sonication for 30 min before centrifugation at 12,000 rpm for 10 min at 4 °C. The supernatant was collected and filtered through a syringe filter (0.22 μm) prior to analysis. 

*Choerospondias axillaris*, *Salvia miltiorrhiza Bunge*, *Syzigium aromaticum*, *Dryobalanops* and *Tabaschir* were processed using the same sample preparation.

#### 3.3.2. Preparation of Plasma Sample and Quality Control Sample

An aliquot of 0.5 mL of plasma was diluted with 50 mL of 4.5% hydrochloride acid and 5 mL of isopropanol, followed by the vortex for 5 min and centrifuged at 12,000 rpm for 10 min at 4 °C. The supernatant was dried under a nitrogen stream and the dryness was re-dissolved with methanol/water (80:20 *v*/*v*) and the above procedure was repeated twice. A volume of 10 μL was injected into the LC-Q-TOF/MS system for analysis.

The appropriate mixed solution containing 18 standards were piped into 100 μL of blank plasmafrom six random rats to generate a pooled quality control (QC) sample using the same manner as described above.

### 3.4. Instrumentation and Conditions

The LC analysis was performed on an Agilent 1260 LC system equipped with a quaternary pump, an on-line degasser, a column temperature controller and a diode array detector. Chromatographic separation was carried out on a Shimpack XR-ODS C_18_ column (75 mm × 3.0 mm, 2.2 μm) (Shimadzu, Kyoto, Japan), which was kept at 30 °C. The mobile phase consisted of 0.1% formic acid water (A) and 0.1% formic acid acetonitrile (B). The gradient program (B%) for analysis was as follows: 0–28 min, 10–95%; 28–30 min, 95–95%; 30–30.01 min, 95–10%; 30.01–33 min, 10–10%. The flow rate was kept at 0.4 mL/min and the injection volume was set at 5 μL.

Mass spectrometry analysis was carried out on the Triple TOF^TM^ 5600 (AB SCIEX, Foster City, CA, USA), a hybrid triple quadrupole time-of-flight mass spectrometer equipped with ESI source operating in both positive and negative ion modes. For the MS conditions, such as the ion source temperature, ion spray voltage, nebulizer gas (gas 1), heater gas (gas 2), curtain gas and collision energy were optimized to obtain maximum sensitivity and higher resolution. The optimal conditions of the MS analysis were as follows: ion spray voltage for ESI^+^ and ESI^−^ were set at 5000 V and −4500 V with an ion source temperature of 500 °C, respectively. The ion source gas1 and gas2 were both set at 50 psi with curtain gas at 30 psi. Declustering potential was set at 90 V, while nitrogen was used as nebulizer and auxiliary gas. The accurate mass and composition for the precursor ions and fragment ions were controlled by the Peak View Software^TM^ V.2.2 (version 2.2, Sciex, Redwood City, CA, USA). The TOF/MS full scan was operated with the mass range of an *m*/*z* of 100–1500.

### 3.5. Analytical Method Assessment

The QC samples were continuously injected to evaluate the stability and repeatability of the mass spectrometry. The relative standard deviation (RSDs) of the retention times and intensities of 18standards were less than 15% in both positive and negative modes. The results indicated that the established method is highly stable and repeatable, which is suitable for analysis.

In vitro, six replicates of the GXSTC samples were measured for technical replicates measured. In vivo, six replicates plasma samples spiked with a mixed solution containing 18 standards were used for the biological replicates measured. 

### 3.6. MS Data Processing

In order to screen and identify the compounds in vitro, the prototype components and their metabolites in vivo, the TOF/MS data files were processed with the multiple data processing approach using AB Sciex PeakView^®^ and MasterView™ software (version 2.2, Sciex, Redwood City, CA, USA).

The identification process and strategy was consisted of on-line data acquisition and the subsequent post-acquisition data processing. There are mainly three steps for the identification strategy. The first step is to obtain a full mass scan spectral and accurate MS/MS data sets. The batch was set up in random with 5% QC samples. Following this, for the subsequent post-acquisition data processing, PeakView^®^ and MasterView™ software provide multiple data processing techniques, including extracted ion chromatography (EIC), MDF, and FF. Mass defect is the difference between the exact mass of the compound and the nearest integral mass. The similar phytocompounds in the same family share similar mother nucleus structures combined with different substituents (such as hydroxyl, formyl, methyl, methoxy and glycosyl). As a matter of fact, the mass defect between the phytocompounds with different substituents and the mother nucleus structure compounds in the same family changed within a narrow range. For the metabolites identification, the mass defect of metabolites is usually only ±50 mDa compared with that of the original drugs. Therefore, the procedure of the MDF approach is to set a filtering reference in the first place based on all the structures of the mother nucleus structure compounds and original drugs reported [31]. Furthermore, due to the interference of endogenous substances and the low concentration level in the plasma, the MDF and EICs approach were especially suitable for the identification of unknown metabolites of certain drugs. Meanwhile, the same fragment ions and the similar fragmentation patterns can be observed in the same family with neutral loss found in glycosides by losing saccharide residuals. The last step is to elucidate the structures of the compounds and metabolites based on the accurate quasi-molecular ion, isotopic pattern, MS/MS fragmentation pattern, relevant drug biotransformation knowledge and RSC Chemspider Database.

The samples in vitro and in vivo were all determined in six replicates with a minimum intensity of 500 and accurate mass measurements within 5 ppm. In vitro, samples were separately compared against five plant extracts to further identify their sources. In vivo, dosed group samples were compared to corresponding control groups to identify prototype components and their metabolites more accurately and reliably. The ions that were present in plasma samples and absent in the correspondent control samples were extracted and identified as the active component in vivo.

## 4. Conclusions

In this paper, a powerful data mining tool, the MDF and FF approaches combined with high resolution mass spectrometry was employed for rapid identification and characterization of the compounds in GXSTC both in vitro and in vivo. A total of 74 compounds (in vitro), 24 prototype components, and 20 metabolites (in vivo) were elucidated and identified by an accurate mass of the quasi-molecular ion, MS/MS fragmentation patterns were based on MDF and FF approaches and related literature. The identification of the active components and the comparison of their metabolites in different modeled rats are reported here for the first time. The compounds in vitro and active components in vivo may be valuable for the further investigation for pharmacodynamics of the formula and new drug development. The strategy presented in this study also provided a reference for the improvement of the quality control, which might offer insights and technical support for investigating the integrative mechanism of GXSTC.

## Figures and Tables

**Table 1:** Identification of compoundsin Guan-Xin-Shu-Tong capsule (GXSTC) based on mass defect filtering (MDF) and fragment filtering (FF) by liquid chromatography quadrupole-time-of-flight mass spectrometry (LC-Q-TOF/MS).

No.	*t*_R_ (min)	Formula	Identified Constituents	Theoretical Molecular Weight (Da)	Measured Mass (Da)		Theoretical Mass Defect Shift (Da)	Fragment Filter (Da)
					[M + H]^+^	[M − H]^−^		
**1**	0.98	C_5_H_7_NO_3_	l-pyroglutamic acid	129.0425	130.0502		0.1022	84.0
**2**	0.98	C_6_H_13_NO_2_	Leucine	131.1729	132.1023		0.1142	86.0
**3**	1.11	C_16_H_12_O_7_	Rhamnetin	316.0583		315.0521	0.1293	133.0
**4**	1.18	C_6_H_8_O_7_	Citric acid	192.0270	193.0343	191.0199	0.0981	129.0
**5**	1.32	C_9_H_11_NO_2_	Phenylalanine	165.1891	166.0868		0.0973	149.0
**6**	4.26	C_15_H_14_O_6_	Catechin/Epicatechin	290.0790		289.0719	0.1398	135.0
**7**	5.47	C_21_H_20_O_12_	Hyperin	464.0955	465.1133		0.2172	301.0
**8**	6.07	C_24_H_26_O_13_	Salviaflaside	522.1374		521.1317	0.2691	135.0
**9**	6.15	C_21_H_20_O_11_	Kaempferol-7-*O*-glucopyranoside	448.1006	449.1025		0.2121	301.0
**10**	6.74	C_20_H_18_O_10_	Salvianolic acid D	418.3509		417.0838	0.1914	175.0
**11**	7.48	C_17_H_14_O_6_	Salvianolic acid F	314.0790		313.0717	0.1398	135.0
**12**	7.87	C_27_H_22_O_12_	Lithospermic acid	538.1112		537.1053	0.2328	323.0
**13**	9.34	C_15_H_10_O_6_	Kaempferol	286.0478		285.0427	0.1086	151.0
**14**	9.79	C_29_H_26_O_12_	Ethyl lithospermic acid	567.4590		565.1355	0.2640	313.0
**15**	10.27	C_29_H_26_O_12_	Dimethyl lithospermic acid	567.4590		565.1355	0.2640	367.0
**16**	14.42	C_15_H_12_O_7_	Dihydroquercetin	304.0583	305.0654		0.1293	153.0
**17**	16.90	C_19_H_18_O_4_	Tanshinone IIB	310.1205	311.1285		0.1608	265.1
**18**	17.95	C_20_H_20_O_5_	Trijuganone B	340.1311	341.1393		0.1815	265.1
**19**	18.81	C_21_H_20_O_4_	Danshenxinkun D	336.1362	337.1422		0.1764	279.1
**20**	19.70	C_18_H_14_O_3_	Methylene tanshiquinone	278.0943	279.1028		0.1245	279.1
**21**	20.45	C_18_H_16_O_3_	Danshenxinkun B	280.1099	281.1180		0.1401	263.1
**22**	20.79	C_20_H_18_O_5_	Methyl tanshinonate	338.1155	339.1235		0.1659	261.1
**23**	21.47	C_17_H_16_O_3_	Danshenspiroketallactone	268.3000	269.1179		0.1401	233.1
**24**	22.48	C_20_H_28_O_2_	Sugiol	300.4351	301.2166		0.2286	233.1
**25**	23.58	C_20_H_30_O_2_	Salviol	302.2246	303.2327		0.2442	261.1
**26**	23.94	C_19_H_16_O_3_	1-Dehydrotanshinone	292.3000	293.1181		0.1401	263.1
**27**	24.67	C_19_H_20_O_2_	1-Dehydromiltirone	280.1464	281.1545		0.1662	263.1
**28**	26.02	C_30_H_48_O_3_	Ursolic acid	456.3603		455.3543	0.3897	221.0
**29**	26.52	C_18_H_32_O_2_	Linoleic acid	280.2403	281.2478		0.2598	149.0
**30**	29.28	C_19_H_24_O_3_	Miltipolone	300.1726	301.1801		0.2025	271.0

**Table 2:** Identification of compounds in GXSTC by LC-Q-TOF/MS in positive and negative ion modes.

No.	*t*_R_ (min)	Formula	Identified Constituents	Measured Mass		Error (ppm)	Source	MS/MS	Confidence Levels
				[M + H]^+^	[M − H]^−^				
**1**	0.65	C_17_H_24_O_11_	Oleoside-11-methyl ester		403.1205	−3.1	c	175, 159	2
**2**	0.83	C_5_H_11_NO_2_	Betaine ^1^	118.0867		3.7	d	72, 58	2
**3**	0.98	C_5_H_7_NO_3_	l-pyroglutamic acid	130.0502		2.4	a/b	84, 56	2
**4**	0.98	C_6_H_13_NO_2_	Leucine	132.1023		2.4	a/b	86	2
**5**	1.11	C_16_H_12_O_7_	Rhamnetin ^1^		315.0521	3.3	c	191, 173.0096	2
**6**	1.12	C_4_H_6_O_5_	Malic acid		133.0151	2.0	a	115	2
**7**	1.18	C_6_H_8_O_7_	Citric acid	193.0343		1.9	a	129	2
					191.0199	0.8	a	173	
**8**	1.32	C_9_H_11_NO_2_	Phenylalanine	166.0868		2.9	a/b	149	2
**9**	1.55	C_4_H_6_O_4_	Succinic acid		117.0180	3.1	a/b	99, 73	2
**10**	1.66	C_7_H_6_O_5_	Gallic acid ^1^		169.0147	2.6	a	125	1
**11**	2.19	C_9_H_10_O_5_	Danshensu ^1^		197.0457	0.4	b	179, 135.0453	1
**12**	2.67	C_7_H_6_O_4_	Protocatechuic acid ^1^		153.0199	3.1	b	109	1
**13**	3.65	C_7_H_6_O_3_	Protocatechuic aldehyde ^1^		137.0252	5.0	b	109, 93.0277	1
**14**	4.22	C_9_H_8_O_4_	Caffeic acid		179.0354	1.9	a	135.0457, 90.9994	1
**15**	4.26	C_15_H_14_O_6_	Catechin/Epicatechin ^1^		289.0719	0.4	a	245.0822, 203.0716	2
**16**	5.47	C_21_H_20_O_12_	Hyperin ^1^	465.1133		2.8	a	301.0438, 149.0808	2
**17**	5.86	C_14_H_6_O_8_	Ellagic acid ^1^		300.9991	0.1	a	255.0299	1
**18**	6.07	C_24_H_26_O_13_	Salviaflaside		521.1317	3.1	b	359.0799, 248.9608	2
**19**	6.15	C_21_H_20_O_11_	Kaempferol-7-*O*-glucopyranoside ^1^	449.1025		1.2	a	301.0707, 205.0035	2
**20**	6.74	C_20_H_18_O_10_	Salvianolic acid D		417.0838	2.4	b	373, 175	2
**21**	6.87	C_27_H_22_O_12_	Salvianolic acid H		537.1053	2.5	b	339	2
**22**	7.48	C_17_H_14_O_6_	Salvianolic acid F ^1^		313.0717	−0.4	b	269, 161	2
**23**	7.87	C_27_H_22_O_12_	Lithospermic acid ^1^	539.1190		0.9	b	521, 323	2
					537.1053	2.5		339, 295	
**24**	8.16	C_16_H_18_O_9_	Chlorogenic acid ^1^		353.0880	0.4	a	190	1
**25**	8.31	C_27_H_22_O_12_	Salvianolic acid I		537.1053	2.5	b	339	2
**26**	8.55	C_28_H_44_O	Ergosterol ^1^	397.3477		2.9	a	301, 205	2
**27**	8.64	C_36_H_30_O_16_	Salvianolic acid B ^1^	719.1614		0.9	b	521, 323	1
					717.1592	1.8	b	519, 321	
**28**	8.77	C_26_H_22_O_10_	Salvianolic acid A ^1^		493.1141	0.1	b	313, 295	1
**29**	8.78	C_26_H_20_O_10_	Isosalvianolic acid C ^1^		491.0986	0.3	b	31, 293	2
**30**	9.34	C_15_H_10_O_6_	Kaempferol ^1^		285.0427	3.0	a	151, 133	2
**31**	9.41	C_18_H_16_O_8_	Rosmarinic acid ^1^	361.0923		1.2	b	163, 145	1
					359.0771	−0.4	b	197, 179, 161	
**32**	9.47	C_15_H_10_O_7_	Quercetin ^1^		301.0354	−0.1	a	177, 151	1
**33**	9.48	C_37_H_32_O_16_	9″-Methyl lithospermate B		731.1646	3.9	b	495, 248	2
**34**	9.53	C_26_H_20_O_10_	Salvianolic acid C ^1^		491.0986	0.3	b	311, 293	1
**35**	9.79	C_16_H_10_O_8_	3,3′-Di-*O*-methylellagic acid ^1^		329.0210	−1.0	a	298	2
**36**	9.79	C_29_H_26_O_12_	Ethyl lithospermic acid ^1^		565.1355	0.5	b	519, 367	2
**37**	10.27	C_29_H_26_O_12_	Dimethyl lithospermic acid		565.1373	3.7	b	367	2
**38**	11.16	C_18_H_16_O_5_	Tanshindiol B	313.1080		2.8	b	295, 267	2
**39**	11.52	C_18_H_16_O_5_	Tanshindiol C	313.1080		2.8	b	295, 267	2
**40**	12.69	C_18_H_16_O_5_	Tanshindiol A	313.1080		2.8	b	295, 267	2
**41**	13.03	C_10_H_12_O_2_	Eugenol ^1^		163.0770	3.3	c	149, 116.9310	1
**42**	13.03	C_10_H_12_O_2_	Ethyl phenylacetate		163.0770	3.3	c	135, 118	2
**43**	13.03	C_9_H_10_O_2_	2-Methoxy-4-vinylphenol ^1^		149.0609	0.3	c	104	2
**44**	14.20	C_16_H_14_O_4_	Isomperatorin	271.0974		3.1	b	243	2
**45**	14.42	C_15_H_12_O_7_	Dihydroquercetin ^1^	305.0654		−0.8	a	245	2
**46**	15.01	C_8_H_8_O_2_	Anisaldehyde	137.0597		1.5	c	undetected	3
**47**	15.57	C_18_H_16_O_4_	Danshenxinkun A	297.1130		2.9	b	261, 233	2
**48**	16.48	C_19_H_18_O_4_	Hydroxytanshinone IIA	311.1285		1.3	b	265	2
**49**	16.90	C_19_H_18_O_4_	Tanshinone IIB	311.1285		1.3	b	293, 283	2
**50**	17.95	C_20_H_20_O_5_	Trijuganone B ^1^	341.1393		2.6	b	281	2
**51**	18.16	C_12_H_14_0_3_	Acetyl eugenol	207.1021		2.4	c	165	2
**52**	18.81	C_21_H_20_O_4_	Danshenxinkun D	337.1422		−3.8	b	297, 279	2
**53**	19.70	C_18_H_14_O_3_	Methylene tanshiquinone ^1^	279.1028		3.5	b	261	2
**54**	20.45	C_18_H_16_O_3_	Danshenxinkun B ^1^	281.1180		2.6	b	263	2
**55**	20.79	C_20_H_18_O_5_	Methyl tanshinonate ^1^	339.1235		2.5	b	279, 261	2
**56**	21.47	C_17_H_16_O_3_	Danshenspiroketallactone ^1^	269.1179		2.2	b	251, 233, 190	2
**57**	22.31	C_19_H_20_O_3_	Cryptotanshinone ^1^	297.1493		2.6	b	279, 251	1
**58**	22.48	C_20_H_28_O_2_	Sugiol ^1^	301.2166		1.2	b	259	2
**59**	22.49	C_19_H_18_O_4_	Furo[3,2-*c*]naphth[2,1-*e*] oxepin-10,12-dione	311.1282		1.3	b	283, 265	2
**60**	22.61	C_18_H_12_O_3_	Tanshinone I ^1^/Isotanshinone I	277.0870		2.5	b	249, 221	1
**61**	23.23	C_18_H_16_O_2_	2-Isopropyl-8-methyl-3,4-phenanthrenedione ^1^	265.1228		1.5	b	223	2
**62**	23.58	C_20_H_3_0O_2_	Salviol	303.2327		2.6	b	285, 133	2
**63**	23.70	C_18_H_14_O_3_	Dihydrotanshinone I ^1^	279.1024		2.8	b	261	1
**64**	23.94	C_19_H_16_O_3_	1-Dehydrotanshinone	293.1181		3.1	b	275, 263	2
**65**	24.50	C_17_H_12_O_3_	Tanshiniactone	265.0682		0.9	b	237, 209	2
**66**	24.67	C_19_H_20_O_2_	1-Dehydromiltirone ^1^	281.1545		3.1	b	253, 223	2
**67**	25.32	C_19_H_18_O_3_	Tanshinone IIA ^1^	295.1321		3.0	b	277, 265	1
**68**	26.02	C_19_H_22_O_2_	Miltirone ^1^	283.1701		3.0	b	253, 241	1
**69**	26.02	C_30_H_48_O_3_	Ursolic acid		455.3543	2.6	b	221, 101	2
**70**	26. 11	C_30_H_48_O_3_	Oleanolic acid		455.3543	2.2	c	undetected	3
**71**	26.32	C_18_H_36_O_2_	Stearic acid		283.2652	3.3	a	undetected	3
**72**	26.52	C_18_H_32_O_2_	Linoleic acid ^1^	281.2478		1.7	a	151, 149	2
**73**	29.28	C_19_H_24_O_3_	Miltipolone	301.1801		0.6	b	271	2
**74**	29.33	C_16_H_32_O_2_	Palmic acid		255.2332	0.8	a	undetected	3

a: *Choerospondias axillaris*; b: *Salvia miltiorrhiza* Bunge; c: *Syzigium aromaticum*; d: *Tabaschir*. ^1^ as the candidates for marker compounds for the quality control in future specification or chromatographic fingerprint common peak attribution of GXSTC. Confidence Level 1: Compounds that matched to reference standards. Confidence Level 2: Compounds that matched to robust spectral or literature. Confidence Level 3: Compounds that classified.

**Table 3:** Identification of the prototype components in rat plasma after oral administration of GXSTC in both positive and negative modes.

No.	*t*_R_ (min)	Formula	Identified Constituents	ESI^+^, *m*/*z*		ESI^−^, *m*/*z*		Error(ppm)	Source	Confidence
				MS [M + H]^+^	MS/MS	MS [M − H]^−^	MS/MS			Levels
**1**	0.85	C_5_H_11_NO_2_	Betaine	118.0867	72,58	116.0717	-	−0.7	c	2
**2**	1.09	C_4_H_6_O_5_	Malic acid			133.0149	115,71	4.3	a	2
**3**	1.39	C_6_H_8_O_7_	Citric acid			191.0207	173,129	4.6	a	2
**4**	1.66	C_7_H_6_O_5_	Gallic acid			169.0147	125	2.6	a	1
**5**	2.29	C_9_H_10_O_5_	Danshensu			197.0457	179,135	0.4	b	1
**6**	2.70	C_7_H_6_O_4_	Protocatechuic acid			153.0199	109	3.1	b	1
**7**	4.30	C_9_H_8_O_4_	Caffeic acid			179.0356	135,91	3.4	a	2
**8**	7.52	C_18_H_16_O_8_	Rosmarinic acid			359.0779	197,179,161	1.6	b	1
**9**	8.16	C_36_H_30_O_16_	Salvianolic acid B	719.1611	521,323	-	-	0.5	b	1
**10**	8.16	C_16_H_18_O_9_	Chlorogenic acid			353.0880	191	0.4	a	1
**11**	9.42	C_16_H_10_O_8_	3,3′-Di-*O*-methylellagic acid			329.0300	289	1.0	a	2
**12**	11.89	C_18_H_16_O_5_	Tanshindiol C	313.1080	295,267			−4.3	b	2
**13**	12.66	C_18_H_16_O_5_	Tanshindiol A	313.1080	295,267			−4.3	b	2
**14**	14.42	C_15_H_12_O_7_	Dihydroquercetin	305.0654	287,245			−0.8	a	2
**15**	16.40	C_19_H_18_O_4_	HydroxytanshinoneA	311.1285	283,265,240			2.1	b	2
**16**	16.97	C_19_H_18_O_4_	Tanshinone II B	311.1285	283,265,240			2.1	b	2
**17**	22.29	C_19_H_20_O_3_	Cryptotanshinone	297.1462	279,251			2.7	b	1
**18**	22.62	C_18_H_12_O_3_	TanshinoneI	277.0870	249,221			3.1	b	1
**19**	23.72	C_18_H_14_O_3_	DihydrotanshinoneI	279.1024	261			2.8	b	1
**20**	25.30	C_19_H_18_O_3_	Tanshinone II A	295.1329	277,265			0.1	b	1
**21**	25.69	C_19_H_22_O_2_	Miltirone	283.1701	253,241,223			−0.7	b	1
**22**	26.32	C_18_H_36_O_2_	Stearic acid			283.2641		−0.5	a	3
**23**	27.44	C_18_H_32_O_2_	Linoleic acid			279.2325	261	−1.6	a	2
**24**	29.32	C_16_H_32_O_2_	Palmic acid			255.2332	231	−2.8	a	2

a: *Choerospondias axillaris*; b: *Salvia miltiorrhiza Bunge*; c: *Tabaschir.* Confidence Level 1: Components that matched to reference standards. Confidence Level 2: Components that matched to robust spectral or literature. Confidence Level 3: Components that classified.

**Table 4:** Identification of metabolites in rat plasma after oral administration of GXSTC in both positive and negative modes.

No.	*t*_R_ (min)	Formula	Identified Constituents	ESI^+^, *m*/*z*		ESI^−^, *m*/*z*		Error (ppm)	Source	Theoretical Mass Defect Shift (mDa)	Metabolite Identification Levels
				MS [M + H]^+^	MS/MS	MS [M − H]^−^	MS/MS				
**1**	1.02	C_15_H_16_O_10_	Dehydrogenate and dehydroxylate danshensu--glucuronide			355.0666	268,257	−1.4	a	0.1758	2
**2**	1.12	C_21_H_18_O_13_	Quercetin-3-*O*-glucoside			477.0665	257,162	−2.1	a	0.2067	3
**3**	2.33	C_13_H_14_O_10_	Protocatechuic acid-glucuronide			329.0514	261,153	−0.1	a	0.1602	2
**4**	2.52	C_9_H_10_O_8_S	Danshensu-sulfate			277.0024	230,173	−0.2	b/d	0.0909	2
**5**	2.82	C_14_H_16_O_10_	Methylated protocatechuic acid-glucuronide			343.0670	175,167,113	−0.4	a	0.1758	2
**6**	3.58	C_15_H_16_O_10_	Caffeic acid-glucuronide			355.0671	179,135	−0.2	a	0.1758	3
**7**	6.04	C_9_H_10_O_3_	Deoxygenate-danshensu			165.0565	147,124	4.6	b/d	0.0933	2
**8**	6.89	C_10_H_10_O_7_S	Dehydrogenate and dehydroxylate methyl danshensu--sulfate			273.0077	193	0.7	b/d	0.0858	2
**9**	7.26	C_25_H_28_O_10_	Hydroxylated cryptotanshinone-glucuronide			487.1632	311	4.5	d	0.2694	2
**10**	8.18	C_25_H_30_O_9_	Cryptotanshinone catechol-glucuronide			473.1837	297	4.0	d	0.2799	2
**11**	8.19	C_18_H_16_O_11_S	Rosmarinic acid-sulfate			439.0340	395,361	−0.1	b/d	0.153	3
**12**	8.23	C_18_H_18_O_8_	Salvianolic acid R			361.0925	239,177	−1.3	d	0.1812	2
**13**	9.33	C_16_H_12_O_7_	Quercetin 3′-methyl ether			315.0524	247	4.3	a	0.1293	3
**14**	10.42	C_19_H_16_O_3_	Methyl-dihydrotanshinone I	293.1172	230,143			−0.2	a	0.1401	2
**15**	10.74	C_39_H_36_O_16_	Trimethyl-salvianolic acid B			759.1925	547,335	−0.8	a	0.3624	2
**16**	10.93	C_22_H_22_O_4_	Methyl-danshenxinkun D	351.1601	199,135			2.7	a	0.1920	3
**17**	12.50	C_20_H_20_O_4_	Methyl-tanshinone IIB/	325.1433	256			−0.7	a	0.1764	2
			Methyl-hydroxytanshinone IIA								
**18**	16.66	C_18_H_16_O_3_	Methyl-danshenxinkun B	295.1351	281,263			−0.4	a	0.1401	3
**19**	18.40	C_10_H_12_O_5_S	Eugenol-sulfate			243.0337	116	1.5	a	0.0912	3
**20**	30.41	C_10_H_12_O_8_S	Methyl danshensu--sulfate			291.0191	211,196	3.7	c	0.1065	2

a: Healthy dosed group, HL dosed, and ABS dosed group; b: Healthy dosed group; c: ABS dosed group; d: HL dosed group. Confidence Level 2: Metabolites that matched to robust spectral or literature Confidence Level 3: Metabolites that classified.

## Supplementary Materials

The following are available online: Figures S1 and S2 and Tables S1 and S2.

## Abbreviations

ABS	acute blood stasis
BPC	base peak chromatogram
EIC	extracted ion chromatogram
FF	fragment filtering
GXSTC	Guan-Xin-Shu-Tong capsules
HL	hyperlipidemic
IDA	information-dependent acquisition
LC-Q-TOF/MS	liquid chromatography quadrupole-time-of-flight mass spectrometry
*m*/*z*	mass-to-charge ratio
MDF	mass defect filtering
MS/MS	Tandem mass spectrometry
Q-TOF/MS	quadrupole-time-of-flight mass spectrometry
QC	quality control
RSD	relative standard deviation
TCM	traditional Chinese medicine
