# Supplementary material for: Comprehensive Identification of Guan-Xin-Shu-Tong Capsule via a Mass Defect and Fragment Filtering Approach by High Resolution Mass Spectrometry: In Vitro and In Vivo Study
Source: Molecules. 2017 Jun 16;22(6):1007. doi: 10.3390/molecules22061007 (PMC6152795; doi:10.3390/molecules22061007)
Supplement: Supplementary file 1 [file molecules-22-01007-s001.pdf]

Supplementary Files

Comprehensive Identification of Guan-Xin-Shu-Tong Capsule via a Mass Defect and Fragment Filtering Approach by High Resolution Mass Spectrometry: In Vitro and In Vivo Study

Table S1. Identification of reference compounds by LC-Q-TOF/MS in positive and negative ion modes.

No.	$t_{R\,(min)}$	Formula	Identified Constituents	Measured Mass $[M + H]^+$	Measured Mass $[M - H]^-$	Error (ppm)	MS/MS
1	1.74	$C_7H_6O_5$	Gallic acid		169.0227	1.6	125
2	2.27	$C_9H_{10}O_5$	Danshensu		197.0465	0.4	179, 135
3	2.75	$C_7H_6O_4$	Protocatechuic acid		153.0207	1.1	109
4	3.73	$C_7H_6O_3$	Protocatechuic aldehyde		137.0260	1.0	109, 93
5	4.30	$C_9H_8O_4$	Caffeic acid		179.0362	0.8	135/90
6	5.94	$C_{14}H_6O_8$	Ellagic acid		300.9999	0.1	255
7	8.24	$C_{16}H_{18}O_9$	Chlorogenic acid		353.0888	0.4	190
8	8.72	$C_{36}H_{30}O_{16}$	Salvianolic acid B	719.1634		0.8	521, 323
					717.1537	1.0	519, 321
9	8.85	$C_{26}H_{22}O_{10}$	Salvianolic acid A		493.1149	0.1	313, 295
10	9.43	$C_{18}H_{16}O_8$	Rosmarinic acid		359.0784	0.9	197, 161
11	9.50	$C_{15}H_{10}O_7$	Quercetin		301.0368	0.9	178, 151
12	9.60	$C_{26}H_{20}O_{10}$	Salvianolic acid C		491.0994	0.3	311, 293
13	13.09	$C_{10}H_{12}O_2$	Eugenol		163.0778	1.3	149, 116
14	22.27	$C_{19}H_{20}O_3$	Cryptotanshinone	297.1416		0.8	279, 251
15	22.61	$C_{18}H_{12}O_3$	Tanshinone I	277.0833		0.9	249, 221
16	23.71	$C_{18}H_{14}O_3$	Dihydrotanshinone I	279.0987		1.0	261
17	25.30	$C_{19}H_{18}O_3$	Tanshinone IIA	295.1254		1.1	277, 265
18	26.04	$C_{19}H_{22}O_2$	Miltirone	283.1664		1.3	253, 241

Table S2. Identification of compounds in GXSTC by LC-Q-TOF/MS in positive and negative ion modes.

No.	t_R (min)	Formula	Identified Constituents	MS/MS
1	0.65	$C_{17}H_{24}O_{11}$	Oleoside-11-methyl ester	175.0765[M-$C_6H_{12}O_9$]$^-$/159.0815[M-$C_6H_{12}O_{10}$]$^-$
2	0.83	$C_5H_{11}NO_2$	Betaine [1]	72.0824[M-CO-H_2O+H]$^+$/58.0674[M-CO-H_2O-CH_3+H]$^+$
3	0.98	$C_5H_7NO_3$	L-pyroglutamic acid	84.0444 [M-CO-H_2O+H]$^+$/56.0131[M-$C_3H_6O_2$+H]$^+$
4	0.98	$C_6H_{13}NO_2$	Leucine	86.0988[M + H − HCOOH]$^+$
5	1.11	$C_{16}H_{12}O_7$	Rhamnetin [1]	191.0560[M-$C_7H_8O_2$-H]$^-$/173.0096[M-$C_7H_{10}O_3$-H]$^-$
6	1.12	$C_4H_6O_5$	Malic acid	115.0037[M-$H_2$0-H]$^-$
7	1.18	$C_6H_8O_7$	Citric acid	129.0192[M-$H_2$0-C0_2+H]$^+$
				173.0086[M-$H_2$0-H]$^-$
8	1.32	$C_9H_{11}NO_2$	Phenylalanine	149.0241[M-NH_2-H]$^+$
9	1.55	$C_4H_6O_4$	Succinic acid	99.0074[M-$H_2$0-H]$^-$/73.0321[M-CO_2-H]$^-$
10	1.66	$C_7H_6O_5$	Gallic acid [1]	125.0244[M-CO_2-H]$^-$
11	2.19	$C_9H_{10}O_5$	Danshensu [1]	179.0354[M-$H_2$0-H]$^-$/135.0453[M-$H_2$0-C0_2-H]$^-$
12	2.67	$C_7H_6O_4$	Protocatechuic acid [1]	109.0299[M- CO_2-H]$^-$
13	3.65	$C_7H_6O_3$	Protocatechuic aldehyde [1]	109.0226[M-CO-H/93.0277[M-CO_2-H]$^-$
14	4.22	$C_9H_8O_4$	Caffeic acid	135.0457[M-CO_2-H]$^-$/90.9994[M-$C_3H_4O_3$-H]$^-$
15	4.26	$C_{15}H_{14}O_6$	Catechin/Epicatechin [1]	245.0822[M-C_2H_4O-H]$^-$/203.0716[M-$C_4H_6O_2$-H]$^-$
16	5.47	$C_{21}H_{20}O_{12}$	Hyperin [1]	301.0438[M-$C_6H_{12}O_5$+H]$^+$/149.0808[M-$C_{15}H_8O_8$+H]$^+$
17	5.86	$C_{14}H_6O_8$	Ellagic acid [1]	255.0299[M-HCOOH-H]$^-$
18	6.13	$C_{24}H_{26}O_{13}$	Salviaflaside	359.0799[M-$C_6H_{10}O_5$-H]$^-$/248.9608[M-$C_{12}H_{16}O_7$-H]$^-$
19	6.15	$C_{21}H_{20}O_{11}$	Kaempferol-7-*O*-glucopyranoside [1]	301.0707[M-$C_6H_{12}O_5$+H]$^+$/205.0035[M-$C_{13}H_8O_5$+H]$^+$
20	6.74	$C_{20}H_{18}O_{10}$	Salvianolic acid D	373.0975[M-CO_2-H]$^-$/175.0410[M-$C_{10}H_{10}O_7$-H]$^-$
21	6.87	$C_{27}H_{22}O_{12}$	Salvianolic acid H	339.0543[M-$C_9H_{10}O_5$-H]$^-$
22	7.48	$C_{17}H_{14}O_6$	Salvianolic acid F [1]	269.0897[M-CO_2-H]$^-$/161.0250[M-$C_8H_8O_3$-H]$^-$
23	7.87	$C_{27}H_{22}O_{12}$	Lithospermic acid [1]	521.1110[M-$H_2$0+H]$^+$/323.0534[M-$C_9H_{12}O_6$+H]$^+$
				339.0543[M-$C_9H_{10}O_5$-H]$^-$/295.0636[M-$C_{10}H_{10}O_7$-H]$^-$
24	8.16	$C_{16}H_{18}O_9$	Chlorogenic acid [1]	190.8346[M-$C_9H_6O_3$-H]$^-$

25	8.31	$C_{27}H_{22}O_{12}$	Salvianolic acid I	$339.0543[M-C_9H_{10}O_5-H]^-$
26	8.55	$C_{28}H_{44}O$	Ergosterol [1]	$301.0438[M-C_7H_{12}+H]^+/205.0031[M-C_{13}H_{20}O+H]^+$
27	8.64	$C_{36}H_{30}O_{16}$	Salvianolic acid B [1]	$521.1079[M-C_9H_{10}O_5+H]^+/323.0553[M-C_{18}H_{20}O_{10}+H^+$
				$519.1006[M-C_9H_{10}O_5-H]^-/321.0433[M-C_{18}H_{20}O_{10}-H]^-$
28	8.77	$C_{26}H_{22}O_{10}$	Salvianolic acid A [1]	$313.0744[M-C_9H_8O_4-H]^-/295.0639[M-C_9H_{10}O_5-H]^-$
29	8.78	$C_{26}H_{20}O_{10}$	Isosalvianolic acid C [1]	$311.0586[M-C_9H_8O_4-H]^-/293.0483[M-C_9H_{10}O_5-H]^-$
30	9.34	$C_{15}H_{10}O_6$	Kaempferol [1]	$151.0035[M-C_8H_6O_2-H]^-/133.0304[M-C_7H_4O_4-H]^-$
31	9.41	$C_{18}H_{16}O_8$	Rosmarinic acid [1]	$163.0383[M-C_9H_{10}O_5+H]^+/145.0284[M-C_9H_{12}O_6+H]^+$
				$197.0471[M-C_9H_6O_3-H]^-/179.0365[M-C_9H_8O_4-H]^-/161.0257[M-C_9H_{10}O_5-H]^-$
32	9.47	$C_{15}H_{10}O_7$	Quercetin [1]	$177.0193[M-C_6H_4O_3-H]^-/151.0037[M-C_8H_6O_3-H]^-$
33	9.48	$C_{37}H_{32}O_{16}$	9″-Methyl lithospermate B	$495.2833[M-C_{12}H_{12}O_5-H]^-/248.9618[M-C_{24}H_{18}O11-H]^-$
34	9.53	$C_{26}H_{20}O_{10}$	Salvianolic acid C [1]	$311.0586[M-C_9H_8O_4-H]^-/293.0483[M-C_9H_{10}O_5-H]^-$
35	9.79	$C_{16}H_{10}O_8$	3,3'-Di-*O*-methylellagic acid[1]	$298.9863[M-C_2H_6-H]^-$
36	9.79	$C_{29}H_{26}O_{12}$	Ethyl lithospermic acid [1]	$519.0997[M-H_20-CO-H]^-/367.0853[M-C_9H_{10}O_5-H]^-$
37	10.27	$C_{29}H_{26}O_{12}$	Dimethyl lithospermic acid	$367.0853[M-C_9H_{10}O_5-H]^-$
38	11.16	$C_{18}H_{16}O_5$	Tanshindiol B	$295.0978[M-H_2O+H]^+/267.1026[M-C_2H_6O+H]^+$
39	11.52	$C_{18}H_{16}O_5$	Tanshindiol C	$295.0978[M-H_2O+H]^+/267.1026[M-C_2H_6O+H]^+$
40	12.69	$C_{18}H_{16}O_5$	Tanshindiol A	$295.0978[M-H_2O+H]^+/267.1026[M-C_2H_6O+H]^+$
41	13.03	$C_{10}H_{12}O_2$	Eugenol [1]	$149.0608[M-CH_2-H]^-/116.9310[M-C_2H_6O-H]^-$
42	13.03	$C_{10}H_{12}O_2$	Ethyl phenylacetate	$135.0439[M-C_2H_4-H]^-/118.9908[M-C_2H_4-H_2O-H]^-$
43	13.03	$C_9H_{10}O_2$	2-Methoxy-4-vinylphenol [1]	$104.9597[M-C_3H_8-H]^-$
44	14.20	$C_{16}H_{14}O_4$	Isomperatorin	$243.1016[M-CO+H]^+$
45	14.42	$C_{15}H_{12}O_7$	Dihydroquercetin [1]	$245.0444[M-C_2H_4O_2+H]^+$
46	15.01	$C_8H_8O_2$	Anisaldehyde	undetected
47	15.57	$C_{18}H_{16}O_4$	Danshenxinkun A	$261.0910[M-H_2O+H]^+/233.0962[M-C_2H_6O+H]^+$
48	16.48	$C_{19}H_{18}O_4$	HydroxytanshinoneⅡA	$265.123[M-C_2H_6O+H]^+$
49	16.90	$C_{19}H_{18}O_4$	TanshinoneⅡB	$293.1179[M-H_2O+H]^+/283.1341[M-CO+H]^+$
50	17.95	$C_{20}H_{20}O_5$	Trijuganone B [1]	$281.1172[M-C_2H_4O_2+H]^+$
51	18.16	$C_{12}H_{14}0_3$	Acetyl eugenol [1]	$165.0546[M-C_3H_6+H]^+$
52	18.81	$C_{21}H_{20}O_4$	Danshenxinkun D	$297.1121[M-C_3H_4+H]^+/279.1016[M-C_3H_4-H_2O+H]^+$

53	19.70	$C_{18}H_{14}O_3$	Methylene tanshiquinone [1]	261.0914[M-H_2O+H]$^+$
54	20.45	$C_{18}H_{16}O_3$	Danshenxinkun B [1]	263.1076[M-H_2O+H]$^+$
55	20.79	$C_{20}H_{18}O_5$	Methyl tanshinonate [1]	279.1024[M-$C_2H_4O_2$+H]$^+$/261.0918[M-$C_2H_6O_3$+H]$^+$
56	21.47	$C_{17}H_{16}O_3$	Danshenspiroketallactone [1]	251.1080[M-H_2O+H]$^+$/233.0955[M-2H_2O+H]$^+$/190.0785[M-2H_2O-CO-CH_3+H]$^+$
57	22.31	$C_{19}H_{20}O_3$	Cryptotanshinone [1]	279.1373[M-H_2O+H]$^+$/251.1417[M-H_2O-CO+H]$^+$
58	22.48	$C_{20}H_{28}O_2$	Sugiol [1]	259.1694[M-C_3H_6+H]$^+$
59	22.49	$C_{19}H_{18}O_4$	furo[3,2-*c*]naphth[2,1-*e*]oxepin-10,12-dione	283.1341[M-CO+H]$^+$/265.1230[M-C_2H_4-CO+H]$^+$
60	22.61	$C_{18}H_{12}O_3$	Tanshinone I [1]/Isotanshinone I	249.0914[M-CO+H]$^+$/221.0957[M-2CO+H]$^+$
61	23.23	$C_{18}H_{16}O_2$	2-Isopropyl-8-methyl-3,4-phenanthrenedione [1]	223.0748[M-C_3H_6+H]$^+$
62	23.58	$C_{20}H_{30}O_2$	Salviol	285.2215[M-H_2O+H]$^+$/133.1006[M-$C_{11}H_{19}$-H_2O+H]$^+$
63	23.70	$C_{18}H_{14}O_3$	Dihydrotanshinone I [1]	261.0912[M-H_2O+H]$^+$
64	23.94	$C_{19}H_{16}O_3$	1-Dehydrotanshinone	275.1071[M-H_2O+H]$^+$/263.0707[M-C_2H_6+H]$^+$
65	24.50	$C_{17}H_{12}O_3$	Tanshiniactone	237.0893[M-C_2H_4+H]$^+$/209.0962[M-C_3H_4O+H]$^+$
66	24.67	$C_{19}H_{20}O_2$	1-Dehydromiltirone [1]	253.1593[M-CO+H]$^+$/223.1118[M-C_4H_{10}+H]$^+$
67	25.32	$C_{19}H_{18}O_3$	Tanshinone IIA [1]	277.1216[M-H_2O+H]$^+$/265.0859[M-C_2H_6+H]$^+$
68	26.02	$C_{19}H_{22}O_2$	Miltirone [1]	253.1222[M-C_2H_6+H]$^+$/241.1224[M-C_3H_6+H]$^+$
69	26.02	$C_{30}H_{48}O_3$	Ursolic acid	221.0837[M-$C_{15}H_{22}O_2$-H]$^-$/101.0025[M-$C_{27}H_{34}O_2$-H]$^-$
70	26. 11	$C_{30}H_{48}O_3$	Oleanolic acid	undetected
71	26.32	$C_{18}H_{36}O_2$	Stearic acid	undetected
72	26.52	$C_{18}H_{32}O_2$	Linoleic acid [1]	151.0283[M-$C_7H_{13}O_2$+H]$^+$/149.0250[M-$C_7H_{15}O_2$+H]$^+$
73	29.28	$C_{19}H_{24}O_3$	Miltipolone	271.1329[M-2CH_3+H]$^+$
74	29.33	$C_{16}H_{32}O_2$	Palmic acid	undetected

[1] As the candidates for marker compounds for the quality control in future specification or chromatographic fingerprint common peak attribution of GXSTC.

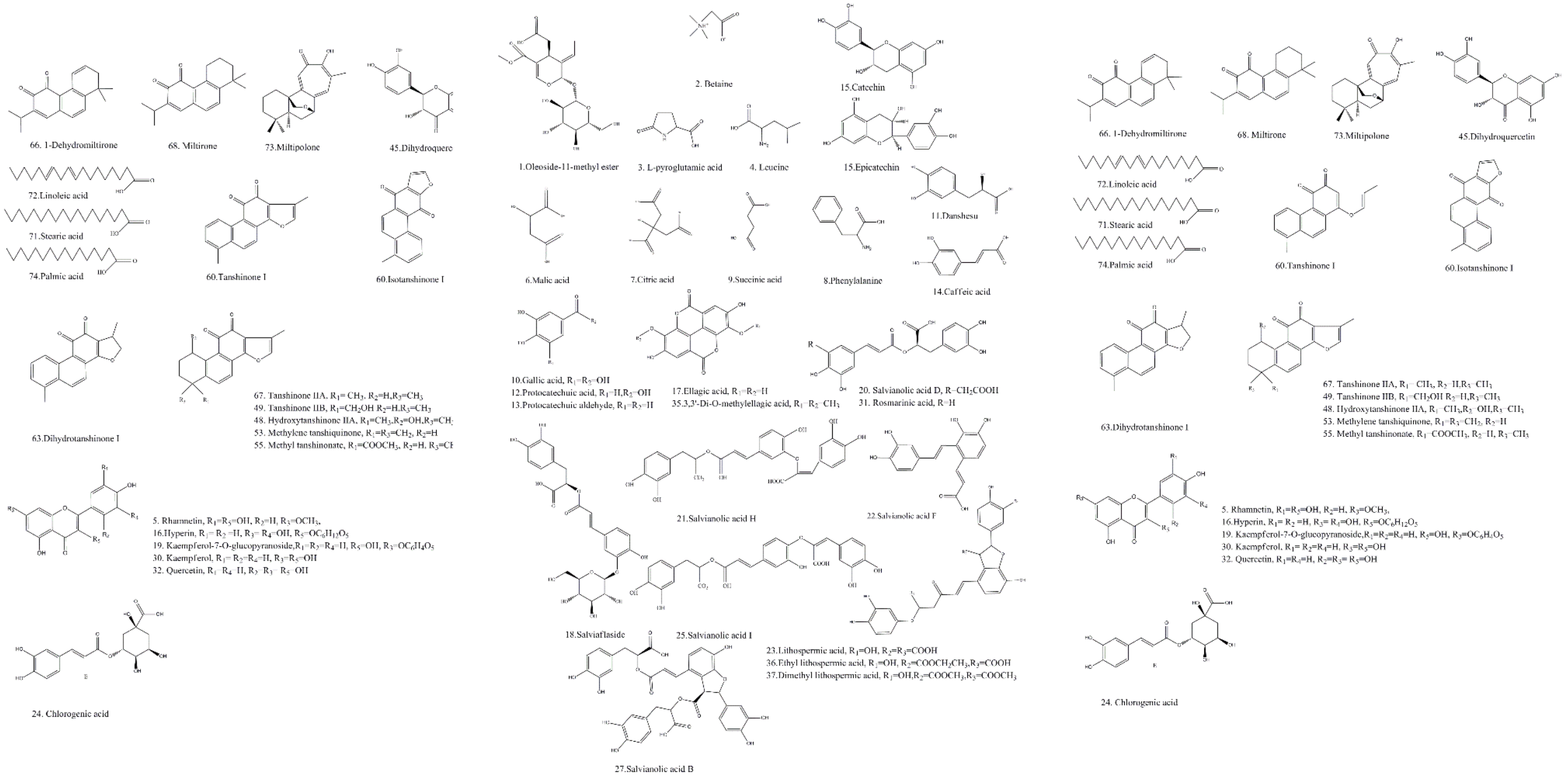

Figure S1. The structures of 74 compounds.

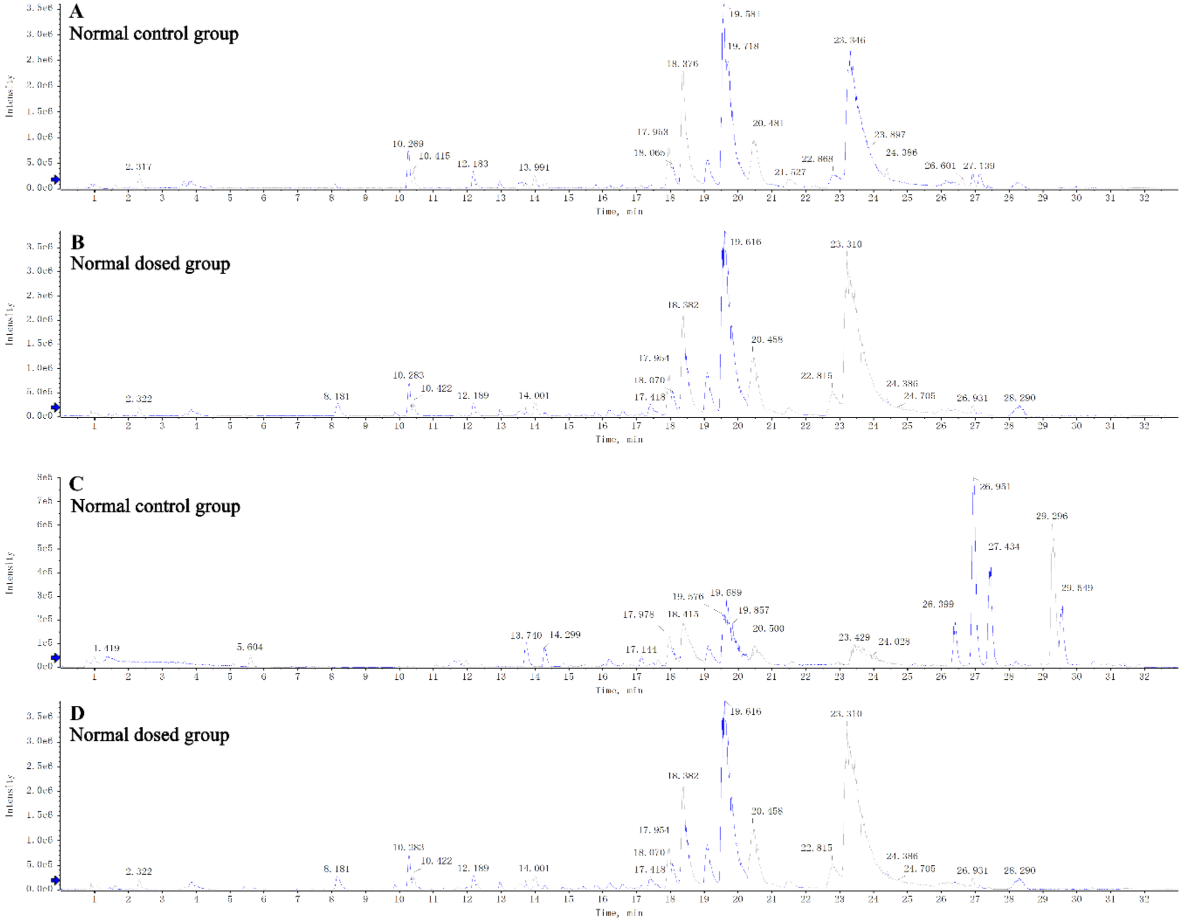

Figure S2. The BPC of normal groups in both modes.

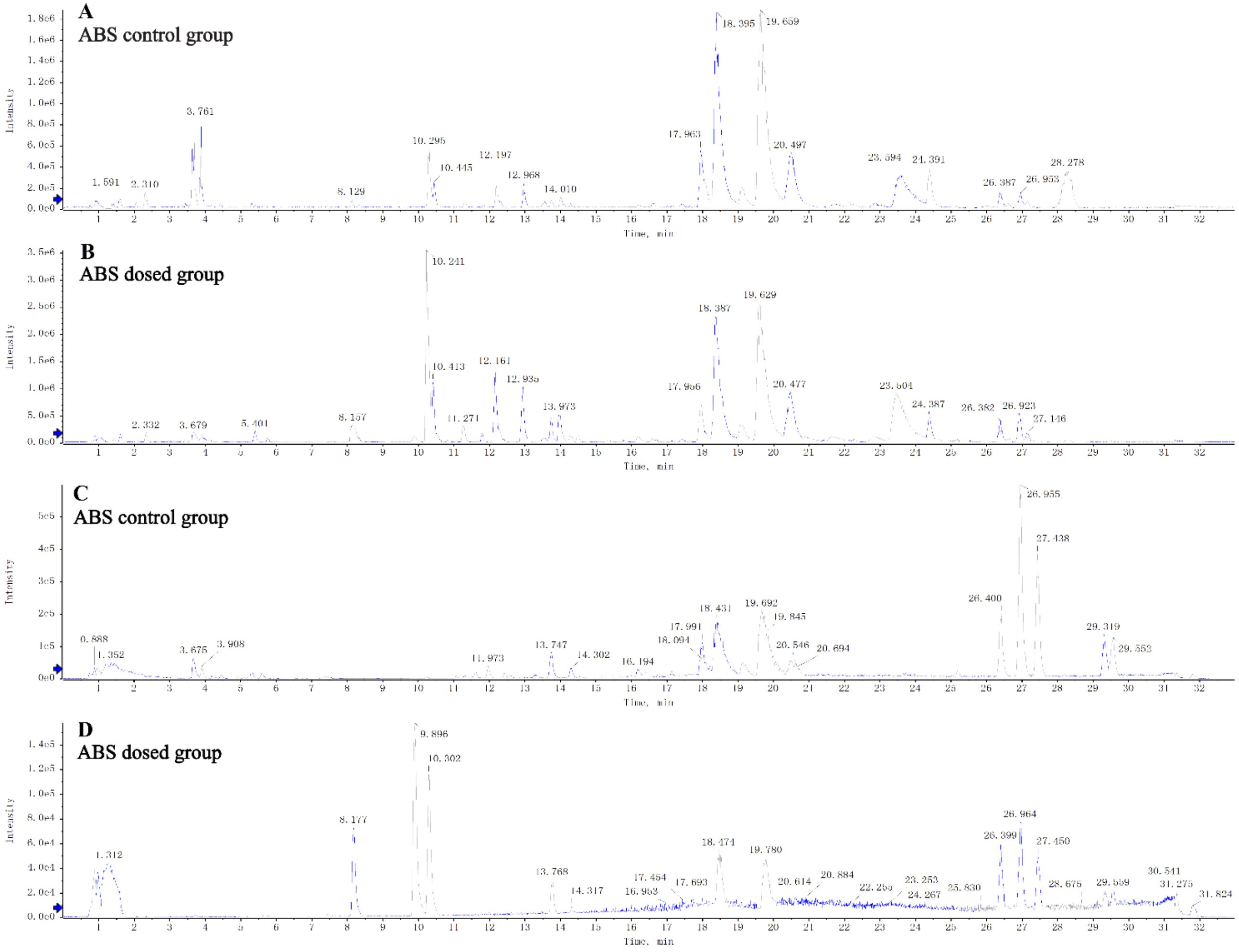

Figure S3. The ABS groups in both modes.

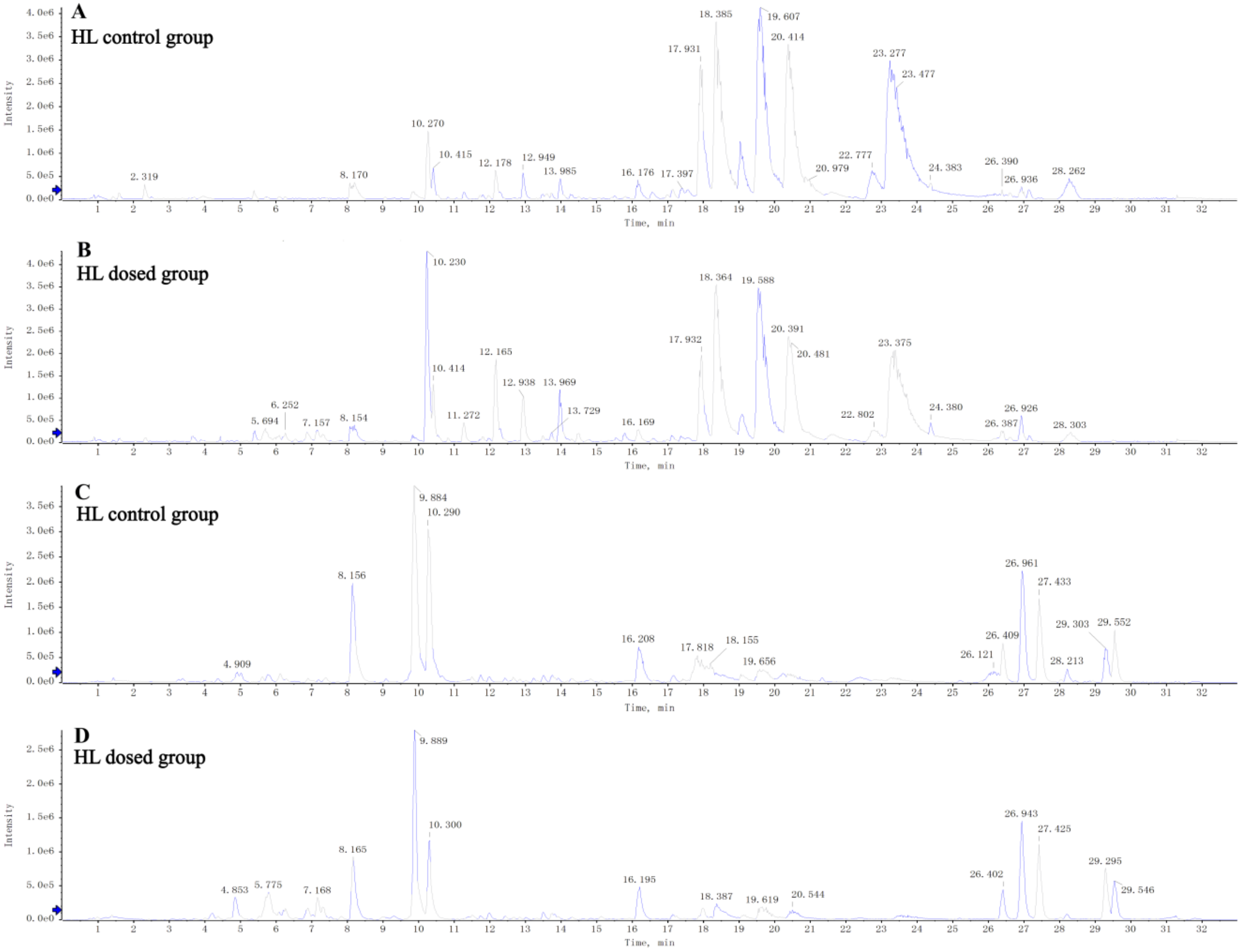

Figure S4. The HL groups in both modes.
